# Epigenome-wide association study and multi-tissue replication of individuals with alcohol use disorder: evidence for abnormal glucocorticoid signaling pathway gene regulation

**DOI:** 10.1038/s41380-020-0734-4

**Published:** 2020-05-12

**Authors:** Falk W. Lohoff, Arunima Roy, Jeesun Jung, Martha Longley, Daniel B. Rosoff, Audrey Luo, Emma O’Connell, Jill L. Sorcher, Hui Sun, Melanie Schwandt, Colin A. Hodgkinson, David Goldman, Reza Momenan, Andrew M. McIntosh, Mark J. Adams, Rosie M. Walker, Kathryn L. Evans, David Porteous, Alicia K. Smith, Jisoo Lee, Christine Muench, Katrin Charlet, Toni-Kim Clarke, Zachary A. Kaminsky

**Affiliations:** 1 Section on Clinical Genomics and Experimental Therapeutics, National Institute on Alcohol Abuse and Alcoholism, National Institutes of Health, Bethesda, MD USA; 2 Royal’s Institute of Mental Health Research, University of Ottawa, Ottawa, Canada; 3 Office of the Clinical Director, National Institute on Alcohol Abuse and Alcoholism, National Institutes of Health, Bethesda, MD USA; 4 Laboratory of Neurogenetics, National Institute on Alcohol Abuse and Alcoholism, National Institutes of Health, Bethesda, MD USA; 5 Clinical Neuroimaging Research Core, National Institute on Alcohol Abuse and Alcoholism, National Institutes of Health, Bethesda, MD USA; 6 Division of Psychiatry, Centre for Clinical Brain Sciences, University of Edinburgh, Edinburgh, UK; 7 Medical Genetic Section, Centre for Genomic and Experimental Medicine, Medical Research Council Institute of Genetics and Molecular Medicine, University of Edinburgh, Edinburgh, UK; 8 Centre for Cognitive Ageing and Cognitive Epidemiology, University of Edinburgh, Edinburgh, UK; 9 Department of Gynecology and Obstetrics, Emory University, Atlanta, Georgia USA; 10 Department of Psychiatry & Behavioral Sciences, Emory University, Atlanta, GA USA

**Keywords:** Diagnostic markers, Addiction

## Abstract

Alcohol use disorder (AUD) is a chronic debilitating disorder with limited treatment options and poorly defined pathophysiology. There are substantial genetic and epigenetic components; however, the underlying mechanisms contributing to AUD remain largely unknown. We conducted the largest DNA methylation epigenome-wide association study (EWAS) analyses currently available for AUD (total *N* = 625) and employed a top hit replication (*N* = 4798) using a cross-tissue/cross-phenotypic approach with the goal of identifying novel epigenetic targets relevant to AUD. Results show that a network of differentially methylated regions in glucocorticoid signaling and inflammation-related genes were associated with alcohol use behaviors. A top probe consistently associated across all cohorts was located in the long non-coding RNA growth arrest specific five gene (*GAS5*) (*p* < 10^−24^). GAS5 has been implicated in regulating transcriptional activity of the glucocorticoid receptor and has multiple functions related to apoptosis, immune function and various cancers. Endophenotypic analyses using peripheral cortisol levels and neuroimaging paradigms showed that methylomic variation in *GAS5* network-related probes were associated with stress phenotypes. Postmortem brain analyses documented increased *GAS5* expression in the amygdala of individuals with AUD. Our data suggest that alcohol use is associated with differential methylation in the glucocorticoid system that might influence stress and inflammatory reactivity and subsequently risk for AUD.

## Introduction

Multiple pathways to the development of alcohol use disorder (AUD) exist and include a complex interplay of environmental and genetic risk factors [1]. Genetic factors have been suggested to play a significant role in the etiology of AUD, as evidenced by twin, family, and adoption studies with heritability estimates ranging between 40 and 60% [2–4]; however, identifying the risk alleles has been difficult due to the complex mode of inheritance, significant clinical and genetic heterogeneity, and large number of genetic variants involved, each only contributing a small fraction to the overall risk.

The field of epigenetics is rapidly developing in AUD and might help explain some of the environmental components as they interact with the genetic architecture [5–8]. Several mechanisms contribute to epigenetic regulation, broadly defined as changes in gene expression without DNA sequence alterations, including histone modifications, non-coding RNA, and DNA methylation changes [9]. It is thought that various epigenetic mechanisms contribute to the pathophysiology of addictions. Some are drug-specific, while others are more generally involved in common pathways that lead to maladaptive and addictive behaviors [1, 10, 11].

While there has been some work on all of these epigenetic mechanisms in AUD, in particular, using various animal models [6, 12, 13], most studies were candidate gene driven and only a few studies used human tissue e.g. ref. [14]. Recent availability of DNA methylation array capture for comprehensive genome-wide profiling, has made it possible to conduct epigenome-wide association studies (EWASs). Only a few EWAS for AUD exist, but they are limited by small sample sizes, low array-capture, tissue types, inconsistent analysis strategy, and data interpretation [15–25]. Consequently, no universal DNA methylation loci for AUD have been identified; however, recent data suggest multiple loci associated with mild-moderate alcohol consumption [26] and interesting new targets for AUD [27].

To address these gaps in the literature, we conducted the largest EWAS analyses currently available for AUD using a cross-tissue/cross-phenotypic approach with the goal of identifying novel epigenetic targets relevant to AUD.

## Materials and methods

### Subjects

**Fig. 1:**
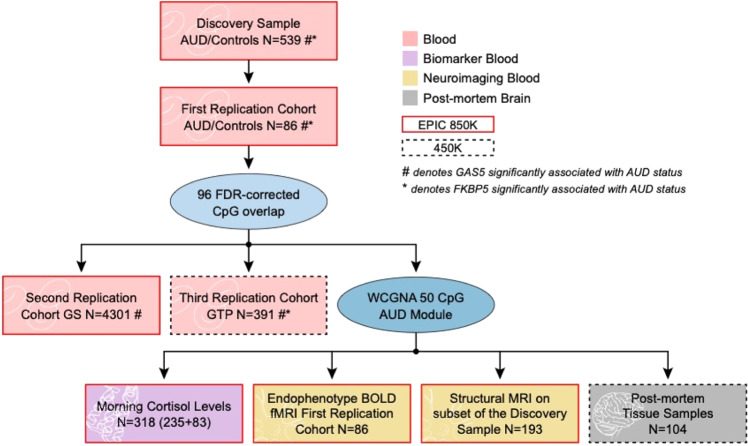
Flowchart of analyses and datasets used. Six independent cohorts were assessed. These included the discovery sample, three replication cohorts, and two post-mortem brain cohorts. AUD alcohol use disorder, FDR false discovery rate, GS Generation Scotland: Scottish Family Health Study, GTP Grady Trauma Project, WCGNA weighted genome coregulation network analysis, BOLD blood oxygen level-dependent, fMRI functional magnetic resonance imaging, MRI magnetic resonance imaging, GAS5 growth arrest specific 5, FKBP5 FK506-binding protein 5.

We used epigenome-wide data from six independent cohorts as follows (see also Fig. 1).

#### Discovery cohort (blood, *N* = 539)

The discovery cohort comprised 539 participants (336 AUD and 203 controls) and was recruited at the National Institute on Alcohol Abuse and Alcoholism (NIAAA) at the National Institutes of Health (NIH), USA. All participants completed the Structured Clinical Interview for Diagnostic and Statistical Manual of Mental Disorders (DSM)-IV-TR (SCID-IV) [28] and an alcohol dependence (AD) diagnosis was determined. A diagnosis of AD in the DSM-IV is equivalent to moderate to severe AUD diagnosis according to the DSM-5 with a concordance of 93% [29]. In this manuscript we use AUD to be consistent with current nomenclature. Subjects completed several self-report questionnaires and clinical assessments. Peripheral blood was obtained for subsequent biomarker and DNA methylation analyses. All participants provided written informed consent in accordance with the Declaration of Helsinki and the study was approved by the Institutional Review Board of the NIAAA. Detailed demographic information can be found in Supplementary Table S1.

#### First replication cohort (blood, *N* = 86)

The first blood replication cohort included 86 participants (43 AUD and 43 controls) recruited to the NIAAA intramural program for a study on fear conditioning and extinction in AUD. Participants were included if they met the following criteria: between 21 and 65 years of age, able to provide written informed consent and cleared for venous access. In the AUD group, participants were also required to have a diagnosis of AD as assessed by the SCID-IV, to specify alcohol as their drug of choice, and to report alcohol consumption within the last 30 days on the Timeline Follow Back (TLFB) [30]. Participants were excluded if in their history and physical examination they reported neurological symptoms of the wrist or arm or reported chronic use of psychotropic medications within 4 weeks, fluoxetine use within 6 weeks, or incidental use of psychotropic medication within 5 half-lives of the beginning of the study. Additional exclusion criteria included presence of ferromagnetic implants, pregnancy, breastfeeding, left-handedness, claustrophobia, magnetic resonance imaging- (MRI-) incompatible intrauterine device or DSM-IV diagnosis of bipolar disorder, psychotic disorder, or current substance dependence other than alcohol, nicotine, or caffeine. Participants not seeking treatment for AUD were excluded if they had a history of alcohol-related seizures or presented with alcohol withdrawal symptom scores ≥ 8 on the Clinical Institute Withdrawal Assessment Alcohol Revised (CIWA-Ar) [31]. All participants provided peripheral blood for various biomarker assessments and DNA methylation studies. All participants provided written informed consent in accordance with the Declaration of Helsinki and the study was approved by the Institutional Review Board of the NIAAA. Detailed demographic information can be found in Supplementary Table S2.

#### Second replication cohort (blood, *N* = 4301)

The second replication cohort was from the Generation Scotland: Scottish Family Health Study (GS), a family-based cohort described elsewhere [32, 33]. Briefly, this cohort includes over 24,000 participants age 18–99 recruited between 2006 and 2011 for no specific disorder across Scotland. Alcohol consumption was assessed at baseline using a pre-clinical questionnaire and participants self-identified as current, former, or never drinkers. Average consumption was a self-report measure reflecting average weekly use in units. A table containing the units of alcohol contained in various drink types was available in order for participants to accurately estimate intake. All components of the GS received ethical approval from the National Health Service Tayside Committee on Medical Research Ethics (REC Reference Number: 05/S1401/89) and written consent was obtained from all participants. DNA methylation data was obtained for 5190 GS individuals from peripheral blood samples taken at baseline using the Infinium MethylationEPIC BeadChip. After quality control, 4301 participants were included in the analyses.

#### Third replication cohort (blood, *N* = 391)

The third blood replication cohort included participants from the Grady Trauma Project (GTP), and details of the sample have been described previously [34–36]. In brief, the GTP was a study on stressful life events and their predictors in a predominantly African American, urban population of low socioeconomic status. The sample included 1561 individuals who were recruited from a primary care clinic where they provided written informed consent, after which they completed a verbal interview and a blood draw. From this cohort, epigenetic information was available for 391 participants (females = 115, males = 276). The SCID-IV was administered and information on lifetime AUD was available for 328 of the 391 participants.

#### Postmortem brain cohorts (*N* = 58 and *N* = 46)

Information on DNA methylation of neural tissues was available from two postmortem cohorts. First, postmortem frontal cortex fluorescence activated cell sorted (FACS) neuronal and glial tissues of 58 individuals with and without major depressive disorder were available for analysis as described previously [37]. Of these 58 individuals, 7 had alcohol use problems (defined retrospectively based on responses to clinical interviews). Second, DNA methylation data on postmortem samples from a cohort of 46 participants with and without alcohol use problems was available from the Gene Expression Omnibus (GSE49393). This dataset included samples from individuals with DSM-IV defined AD (*n* = 9), alcohol abuse (*n* = 14), as well as an age-matched healthy control group (*n* = 23). For our study, we combined data from participants with AD and alcohol abuse to define a new group, ‘AUD’ (*n* = 23). Sample collection and processing for this cohort have been described in a previous publication [38].

### Methylomic profiling

DNA methylation data from whole blood samples were assessed using an Infinium MethylationEPIC BeadChip microarray (Illumina Inc., San Diego, California) according to the manufacturer’s protocol for the discovery, first, and second replication cohorts (NIAAA and GS). The third replication sample (GTP) and neural tissues from both postmortem cohorts were assessed with the Illumina 450K chip as described previously [37, 38]. Pre-processing of the GS data has been described in detail elsewhere [39].

### Statistical analysis

Raw data from discovery and replication cohorts were processed with the package ‘wateRmelon’ in R [40]. All cross-reactive probes and probes failing quality assessment were removed. Scale-based correction was applied to Illumina type I versus type II probes. Methylated and unmethylated intensities were quantile-normalized in the red and green channels separately using the Dasen method in WateRmelon [40], followed by *β*-value (intensity ratios of methylated to unmethylated probes) calculation. All analyses were carried out in R, version 3.5.1 (© 2018 The R Foundation for Statistical Computing).

Linear regressions were used to examine the associations of each CpG site with AUD in the discovery and first replication cohort. DNA methylation beta values were regressed on AUD status adjusting for age, sex, race, and cell type composition of blood. Cell type composition was derived from DNA methylation proxies using the Houseman algorithm [41]. Significant probes (*p* ≤ 0.05) in both the discovery and first replication cohorts were identified and further examined as follows.

All probes identified above were tested for associations with AUD diagnosis in the second (GS) and third (GTP) blood replication cohorts. Associations were tested in the GTP cohort using linear regressions as described above. In the GS cohort, linear regression models were fit in the *limma* R packages with CpG site as an outcome variable and log transformed units per week as an independent variable. Age, sex, smoking status, pack years of smoking, and the first 20 principal components from the M-values corrected for age, sex, relatedness batch, and estimated cell counts were fit as covariates in the linear model.

#### Postmortem brain analysis

Methylation levels of the target probes were regressed on alcohol use status controlling for age, sex, and race (only in the first postmortem set), either separately for neuronal and glial tissue (first postmortem dataset) or adjusting for neural cell type proportion (second postmortem dataset).

#### Weighted genome coregulation network analysis (WGCNA)

WGCNA was performed to detect clusters (modules) of highly correlated genes using the top 96 probes exhibiting consistent FDR significance with AUD diagnosis from the discovery and replication cohorts. Module detection was carried out using a soft thresholding power β of 9, which was chosen by maximizing scale free topology model fitting as a function of model connectivity based on the internal data structure according to the recommendations of Horvath [42]. All analyses were performed in R.

#### Mediation network analysis

A mediation analysis approach was performed on WGCNA identified module loci such that each loci in the top 30th percentile of module membership or ‘hubness’ is assessed for association to the outcome metric (cortisol, see results), both alone and in conjunction with every other module locus to assess for mediation. Those connections implicated as mediating by means of statistical association below 5% with the outcome alone and no association in an additive model are considered ‘connected’. Connected genes are quantified and displayed using the iGraph package in R.

### Endophenotype analysis

#### Cortisol measurements and assessments

All blood samples were taken before 09:00 AM after inpatient admission. Cortisol was measured using radioimmunoassay (RIA) with an intra-assay and inter-assay coefficient of variation of 3.5% and 14.3%, respectively.

#### BOLD fMRI of fear conditioning and extinction (brain; *N* = 86)

Subject description provided under first replication cohort.

*Fear conditioning task*: The Fear Conditioning and Extinction (FCE) experiment took place after individuals with AUD had no withdrawal symptoms for at least two consecutive days. Day 1 consisted of habituation, conditioning, and two extinction blocks and Day 2 consisted of extinction recall and renewal blocks. Galvanic skin responses were obtained throughout the fMRI session using Ag/AgCl electrodes (41 mm diameter). Two additional electrodes were placed on the wrist of the same hand to deliver electrical stimulation (unconditioned stimulus [US]; 0.5 s duration). The intensity level of electrical stimulation was uncomfortable but tolerable as determined by a personalized work-up on the first day. Participants were presented with digital photographs of two different rooms, each containing a lamp shade that turned blue or yellow as the conditioned stimuli (CSs). During conditioning, one of the colors was paired with a 0.5-s electrical stimulation (CS+) in 75% of the trials, while the other was never followed by a shock (CS−). For further details please see [43].

*fMRI data processing*: Functional data were processed using Statistical Parametric Mapping (SPM12b, Wellcome Department of Cognitive Neurology, London, UK) based on MATLAB R2018b (MathWorks, Natick, MA, USA). After removal of the first three individual functional scans of the experimental conditioning phase to avoid artefacts caused by magnetic saturation effects, and prior to preprocessing, all images were visually controlled for gross movement artefacts and anatomical abnormalities; all images of the 86 subjects were included. Scans were further corrected for signal‐to‐noise decrease in single slices, using the denoising function of the ArtRepair software (http://cibsr.stanford.edu/tools/human-brain-project/artrepair-software.html, assessed 03 Jan, 2019). Afterwards, individual scans were spatially realigned to correct for head motion and normalized using the warping parameters estimates of the individual co-registered and segmented MPRGE image. Images were normalized to an isovoxel size of 3.5 × 3.5 × 3.5 mm. Subsequent smoothing was done using an isotropic Gaussian kernel (8 mm FWHM).

The preprocessed fMRI data were then analyzed as a block design for the conditioning phase in the context of the general linear model approach using a two‐level procedure. On the individual single subject level, the different conditions (CS+ and CS−) were modeled (boxcar functions convolved with the hemodynamic response function) as explanatory variables together with the six movement parameters to account for residual variance due to head motion, and a single constant representing the mean over scans. Both CS+ and CS− conditions in the fear conditioning phase were modeled as separate regressors for trials 2–20 since learning of CS+ (potential shock) versus CS- (no shock) has not occurred yet during the first trial. Subsequently, for each subject, linear contrast images were computed for fear conditioning: ‘CS+ minus CS−‘, trials 2–20. For these individual contrast images, overall blood oxygenation level-dependent (BOLD) responses were assessed for the “effect of interest” provided by an analysis of variance. Here, we specifically assessed small volume adjusted BOLD responses of anatomical atlas-based a priori Regions of Interest (ROI) as brain regions crucially involved in emotion detection and regulation, i.e., left/right amygdala, left/right hippocampus, left/right medial prefrontal cortex (mPFC) (i.e., aal-mask for Front_Med_Orb), left/right insula, left/right rostral anterior cingulate cortex (rACC, ROI as defined in a prior study [44], using the WFU PickAtlas toolbox (http://fmri.wfubmc.edu/software/PickAtlas). Parameter estimates of the ROI BOLD responses (identifying clusters with an initial voxel-level threshold of *P *(uncorrected) < 0.005) were then extracted from the cluster peak as SPM eigenvariates for further BOLD analyses as described below.

*BOLD analysis:* Associations between blood methylation levels for our target probes and BOLD signal data (from the first replication cohort) were examined. As the normality assumption for residuals was not met for regressions of methylation on BOLD signal, we used non-parametric Kendall’s partial correlations. For this, we regressed out inter-individual differences in cell type composition and estimated the correlations of the residuals with BOLD signal, adjusting for age, sex, and race.

#### Structural MRI (brain; *N* = 193)

A subset of participants from the discovery cohort also participated in a structural MRI study. Hippocampus volumes were determined using the standard Freesurfer [45] (version 5.3.0; surfer.nmr.mgh.harvard.edu) pipeline. The individual T1-weighted images were automatically segmented to measure gray matter volume of structures [46] using the following steps: (1) The images were resampled to 1 mm^3^ voxels; transformed to Talairach space, the intensity non-uniformity corrected [47]; and skull was stripped from the images [48]; and finally auto-segmentation proceeded with labels assigned based on probabilistic location of structures. We conducted a reliability test by examining a random number of the auto-segmented volumes with recon_checker from FreeSurfer’s QATools. This included checking for outliers, calculating signal-to-noise ratio, and visually examining generated snapshots of brain volume segmentation.

#### Targeted human postmortem brain mRNA analyses (brain; *N* = 24)

Postmortem tissues were obtained from the New South Wales Tissue Resource Centre (NSWBTRC) at the University of Sydney, Australia. Brain tissues from 11 males with AUD and 13 controls were analyzed for amygdala and prefrontal cortex (PFC). All AUD subjects had alcohol detected in blood and were also daily smokers at the time of death. Total RNA was extracted from male postmortem frozen brain tissue, using the RNeasy Lipid Tissue mini Kit (Qiagen). One µg total RNA was reverse-transcribed using SuperScript® III First-Strand Synthesis SuperMix for qRT-PCR (Invitrogen). Real-time quantitative polymerase chain reaction (PCR) was run in ViiA™ 7 Real-Time PCR System using TaqMan Gene Expression Assays (GAS5; Hs03464472_m1, Thermo Fisher). Data and statistical analyses were performed using GraphPad prism 8.0 (GraphPad Software Inc., San Diego, CA, USA). An unpaired *t*-test was used to determine statistical significance with *p* < 0.05.

## Results

### Associations of DNA methylation in blood with AUD status

**Fig. 2:**
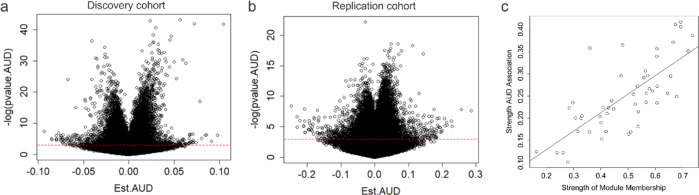
Volcano plots of DNA methylation association with AUD. Volcano plots depicting the effect sizes of DNA methylation association with AUD (x axis) as a function of the negative natural log of the *p* value (*y* axis) for the discovery (**a**) and replication (**b**) cohorts. Red dashed horizontal line depicts a nominal *p* value of 0.05. **c** Scatter plot showing the relationship between module membership of probes (*x* axis) versus associations with AUD status (*y* axis).

We identified 69242 CpG probes that were significantly (*p* ≤ 0.05) associated with AUD in the discovery cohort and 72941 probes in the first replication cohort, using a linear regression model additively controlling for age, sex, and race as covariates. Demographic and sample characteristics can be found in Supplementary Tables S1 and S2. After false discovery rate (FDR) correction for multiple testing (FDR threshold *p* < 0.05), 5101 probes in the discovery and 203 probes in the first replication sample remained significant (Figs. 1, 2a, b). Of these, 96 probes were consistently FDR significant in both cohorts (notably all in the same direction), representing an overlap significantly higher than that expected by chance (expected probability= 0.0006; 95% CI = 0.40–0.54) (Supplementary Table S3). To further refine this set, we performed additional replication analyses using linear models for quantitative alcohol consumption in Generation Scotland: the Scottish Family Health Study (GS:SFHS) and SCID lifetime AUD diagnosis in the Trauma Project (GTP) (see Supplementary Tables S4, S5). Of the 96 originally identified loci, a total of 70 exhibited significant (*p* < 0.05) associations in at least one of the two cohorts. Notably, two probes located within the long non-coding RNA growth arrest specific 5 gene (*GAS5)* (*GAS5*a = cg06644515, *GAS5*b = cg16290996) were among the CpGs most consistently associated with AUD or alcohol use across all cohorts investigated (Table 1).

**Table 1:** Association of DNA methylation with AUD status across multiple cohorts.

		Discovery Cohort (*N* = 539)			Replication Cohort (*N* = 86)			Generation Scotland (*N* = 4301)			Grady Trauma Project (*N* = 391)		
Probe ID	Gene	*b*	SE	Adj *P*	*b*	SE	Adj *P*	*b*	SE	Adj *P*	*b*	SE	Adj *P*
*Negative Associations*													
cg06644515	GAS5a; SNORD47	−0.015	0.003	0.00054	−0.044	0.0071	0.0028	−0.017	0.0017	3.00E-24	−0.022	0.0064	0.00071
cg15130459	SNORD72;RPL37	−0.016	0.0044	0.04	−0.044	0.0089	0.033	−0.0062	0.0023	0.0079	−0.025	0.0085	0.0044
cg20813374	FKBP5	−0.02	0.0036	0.00014	−0.045	0.0091	0.03	−0.0032	0.002	0.12	−0.014	0.0054	0.011
cg01940273		−0.056	0.006	0.006	−0.078	0.013	0.0028	−0.011	0.0026	0	−0.023	0.009	0.012
cg14316231	MYST3	−0.012	0.003	0.028	−0.04	0.0083	0.038	−0.0046	0.002	0.023	−0.014	0.0058	0.018
cg21566642		−0.072	0.0078	0.008	−0.11	0.017	0.0025	0.0032	0.0025	0.2	−0.028	0.012	0.023
cg16290996	GAS5b; SNORD78	−0.014	0.0031	0.0039	−0.043	0.0076	0.0089	−0.014	0.0025	0	−0.018	0.0078	0.025
cg18917643		−0.027	0.0036	0.004	−0.057	0.01	0.0098	−0.015	0.0023	0	−0.013	0.0061	0.034
cg00505318	UBA3	−0.012	0.0022	0.00037	−0.026	0.0053	0.03	−0.0054	0.0018	0.0024	−0.018	0.0088	0.037
cg10558233		−0.026	0.0053	9.00E-04	−0.066	0.013	0.031	−0.0083	0.0032	0.0095	−0.022	0.01	0.039
cg27537125		−0.013	0.0019	0.002	−0.024	0.0045	0.012	NA	NA	NA	−0.009	0.0043	0.039
cg15705813		−0.021	0.0029	0.003	−0.038	0.0063	0.0039	−0.013	0.0018	0	−0.014	0.007	0.046
cg25537245	LINC01126	−0.0061	0.0014	0.01	−0.016	0.0035	0.049	−0.0072	0.0021	0.00046	−0.0052	0.003	0.085
cg18159646	SNORD22;SNHG1	−0.0082	0.0022	0.034	−0.029	0.0052	0.0099	−0.011	0.0015	3.00E-12	−0.0094	0.0062	0.13
cg02583484	HNRNPA1	−0.03	0.0033	0.003	−0.048	0.0085	0.0093	−0.013	0.0019	0	−0.0089	0.0068	0.19
cg03603381	RASGRP1	−0.015	0.003	0.00052	−0.038	0.0079	0.038	−0.0056	0.0015	0.00011	−0.0072	0.0056	0.2
cg25615944	RPL37	−0.011	0.0028	0.003	−0.035	0.0065	0.012	−0.02	0.0029	0	−0.0084	0.0084	0.32
cg15408734		−0.0075	0.0015	0.00065	−0.02	0.0038	0.014	−0.0064	0.0023	0.0062	−0.003	0.003	0.33
cg18696027	B2M	−0.0087	0.0021	0.011	−0.027	0.0047	0.0089	−0.0067	0.0024	0.005	0.0016	0.0061	0.79
cg20625334	STAM2	−0.026	0.004	0.004	−0.061	0.012	0.023	−0.012	0.0023	0	−0.002	0.0078	0.8
cg22158648		−0.012	0.0028	0.0091	−0.035	0.007	0.03	−0.0062	0.0018	0.00048	0.001	0.007	0.88
cg13683827	FAM113B	−0.01	0.0023	0.0054	−0.032	0.0059	0.012	−0.0069	0.0016	0	−4.00E-04	0.005	0.94
cg05575921	AHRR	−0.1	0.011	1.50E-13	−0.14	0.024	0.0037	−0.016	0.0048	0.00059	NA	NA	NA
*Positive Associations*													
cg08109625	PRKCZ	0.011	0.003	0.044	0.036	0.007	0.023	0.0017	0.0017	0.3	0.015	0.0068	0.027
cg20005742		0.02	0.0049	0.014	0.064	0.013	0.038	0.0099	0.0026	1.00E-04	0.016	0.0079	0.049
cg14334350	NOTCH1	0.017	0.0034	0.00072	0.044	0.0078	0.0089	0.0098	0.0019	0	0.011	0.0064	0.077
cg04406114	POU2F2	0.0097	0.0021	0.0034	0.024	0.0048	0.034	0.0052	0.0015	0.00053	0.0087	0.0062	0.16
cg11095027	TOLLIP	0.024	0.0032	6.58E-09	0.033	0.0067	0.033	0.0081	0.0015	3.00E-8	0.0053	0.0069	0.45
cg09975715	MYH10	0.019	0.0033	3.75E−05	0.042	0.0084	0.028	0.0045	0.0018	0.013	0.0022	0.0047	0.64
cg15554126	PEX14	0.011	0.0029	0.041	0.036	0.0063	0.0089	0.0073	0.0017	0	-0.002	0.0043	0.65
cg11589723	GLTSCR1	0.011	0.0023	0.00065	0.031	0.0065	0.045	0.013	0.0028	0	-0.00075	0.0023	0.75

As smoking is commonly comorbid with AUD and also leads to DNA methylation changes, we carried out additional regressions to test if adjusting for smoking altered our results for the 96 target probes. Methylation at each of the 96 probes was regressed on AUD status, controlling for smoking (self-reported scores on the Fagerstrom’s test for nicotine dependence) in addition to age, sex, race, and cell type. All probes retained significant associations after adjusting for smoking scores (Supplementary Table S6).

In order to address possible dose/life-time alcohol exposure on methylation, we carried out additional exploratory analyses in the discovery cohort. A metric of exposure was calculated by multiplying the average number of drinks per day by the number of years since their self-reported age of drinking inception. Of the 96 CpG sites, 65 probes exhibited significant associations with dose (Supplementary Table S7), suggesting that the observed epigenetic associations in these cases may be a consequence of alcohol exposure. Subset analyses for DNA methylation changes by ethnicity and gender are shown in Supplementary Fig. 1 and Supplementary Table S8.

### Weighted genome coregulation network analysis

Given that methylomic variation is often correlated across CpG sites, we conducted a Weighted Gene Coregulation Network Analysis (WGCNA) adjusting for age, sex, and ethnicity in the 96 probes significantly associated in both the discovery and replication cohorts. We identified one significant AUD associated module consisting of 50 probes (Module association Rho= 0.76, *P* = 2.2 × 10^−16^, Supplementary Table S9), containing glucocorticoid signaling associated genes *GAS5b*, and *FKBP5*, inflammatory cytokine driving genes *LURAP1L* and *LURAP1L-AS1*, and a number of small nucleolar RNA (snoRNA) targets, among others.

### Gene ontology analysis

A gene ontology analysis identified a number of significant biological pathways, including some potentially indicative of dysregulated inflammatory processes (Supplementary Table S10).

### Association of top AUD associated loci and coregulated modules with cortisol

**Fig. 3:**
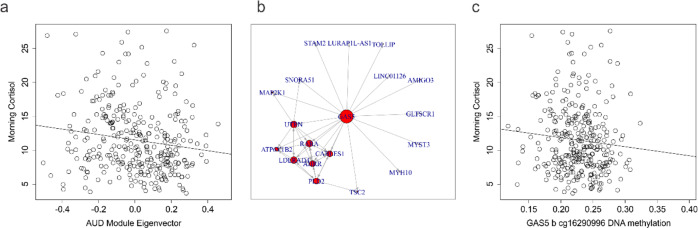
HPA axis metric associations with AUD associated DNA methylation. **a** Scatterplot of morning cortisol (*y* axis) as a function of AUD associated coregulated module eigenvector values (*x* axis). **b** Network diagram from the network mediation analysis demonstrating coregulated genes associated with morning cortisol in the discovery dataset. **c** Scatterplot of morning cortisol (*y* axis) as a function of *GAS5b* DNA methylation (*x* axis).

Based on the WCGNA analyses which revealed top targets relevant to the glucocorticoid system, we investigated DNA methylation associations with morning cortisol (Fig. 3a). Morning cortisol levels collected under the same protocol were available across the discovery and first replication cohorts and were subsequently combined and assessed for associations with AUD coregulated module variation. The first eigenvector of a principal component analysis was generated for those 50 loci in the significant AUD associated module identified by WGCNA in order to represent the majority of this coregulated epigenetic variation. A significant association with morning cortisol levels was observed after adjusting for age, sex, and ethnicity (*b* = −3.85 ± 1.63, *F* = 5.14, df= 4/309, *p* = 0.019) (Fig. 3). To assess module gene functional importance to cortisol variation, we employed a mediation network analysis approach to identify loci with the highest number of cortisol associated connections among coregulated genes. Of those 50 loci interrogated, *GAS5b* exhibited the most evidence for connectedness in association to cortisol (*N* = 18) (Fig. 3b). In light of its functional relationship with HPA axis regulation, we assessed the association between two of the GAS5 probes and observed a significant association with GAS5b (*b* = ‒17.87 ± 8.62, *F* = 4.70, df = 4/309, *p* = 0.039) but not GAS5a (data not shown) (Fig. 3b).

### Association of DNA methylation with stress-related AUD neuroimaging endophenotypes

**Fig. 4:**
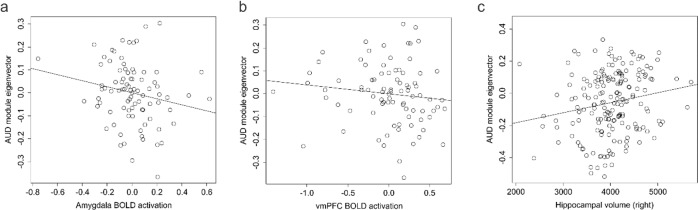
Brain phenotypes as a function of AUD module variation. **a** A scatterplot of amygdala BOLD activation (x axis) as a function of AUD associated module eigenvector variation (*y* axis) in the fear cohort. **b** A scatterplot of vmPFC BOLD activation (*x* axis) as a function of AUD associated module eigenvector variation (*y* axis) in the fear cohort. **c** A scatterplot of right hippocampal volume (*x* axis) as a function of AUD associated module eigenvector variation (*y* axis) in the discovery cohort.

We assessed BOLD activation during fear acquisition to assess how DNA methylation associated with AUD was associated with BOLD signal in different brain regions. Significant associations (*p* ≤ 0.05) were observed after adjusting for age, sex, and race in the left amygdala, and both the left and right insula (Table 2 and Supplementary Table S10). We next assessed associations with morning cortisol and identified significant associations in both the left and right vmPFC, and the right ACC (Table 2). As the AUD associated coregulated DNA methylation module above was associated with cortisol, we investigated the association of the eigenvector with BOLD activation levels and observed significant associations in the right amygdala and a non-significant trend for association with the left vmPFC (Fig. 4a, b). Individual results for the original 96 probes are reported in Supplementary Table S10. Together, the results suggest that DNA methylation changes in response to alcohol exposure have the potential to mediate altered brain activity patterns through alteration of HPA axis activity and stress sensitivity.

**Table 2:** Brain activity associations with AUD phenotypes.

Brain region BOLD	AUD beta	*P* value	Cortisol Tau	*P* value	AUD eigenvector Tau	*P* value
Amygdala (left)	−0.09	0.028	−0.051	0.51	0.028	0.71
Amygdala (right)	0.035	0.49	−0.089	0.24	−0.16	0.032
Hippocampus (left)	−0.049	0.23	–0.085	0.27	0.046	0.53
Hippocampus (right)	−0.018	0.6	−0.14	0.075	−0.015	0.84
Insula (left)	0.13	0.036	0.084	0.27	−0.037	0.61
Insula (right)	0.15	0.039	0.052	0.5	−0.073	0.32
Rostral ACC (left)	0.066	0.17	−0.12	0.11	−0.058	0.43
Rostral ACC (right)	0.088	0.34	−0.18	0.017	−0.018	0.8
vmPFC (left)	0.12	0.23	−0.2	0.0093	−0.12	0.093
vmPFC (right)	0.077	0.21	−0.21	0.0063	−0.076	0.3

### Association of AUD DNA methylation module eigenvector with hippocampal structure

In light of the observed AUD associations with epigenetic regulation of cortisol signaling and previous work demonstrating associations of cortisol with limbic cortical volume [49], we investigated available structural measures with AUD associated epigenetic variation. In the discovery sample, the AUD associated coregulated module eigenvector was significantly associated with right, but not left hippocampal volume after adjusting for age, sex, and race (right β = 546.55 ± 200.021, *F* = 7.103, df = 5/177, *p* = 0.0069; left β = 395.528 ± 219.9, *F* = 3.97, df = 5/177, *p* = 0.074) (Fig. 4c).

### Association of AUD and DNA methylation in *postmortem* tissue

Both *PRKCZ* and *GAS5* were consistently identified in two independent brain tissue cohorts, suggesting these may be among the most robust brain related findings identified. Of the 96 target probes identified in the discovery and first blood replication datasets, only 27 were available for examination in the NICHD postmortem brain dataset (due to differences in the array platforms EPIC 850K versus 450K). In FACS isolated glial tissue, alcohol use status (*N* = 29 cases, *N* = 29 controls) was associated with *PRKCZ* methylation (Supplementary Table S11). In neuronal tissue, alcohol use status (*N* = 29 cases, *N* = 29 controls) was associated with methylation levels of *GAS5*, *GLTSCR1*, and *B2M* (Supplementary Table S11). Methylation data were available for 34 of the 96 target probes in the Australian Brain Bank postmortem brain dataset (*N* = 23 cases, *N* = 23 controls) (GSE49393). DNA methylation of probes located within *UBA3, CABLES1, MYST3, GAS5, MYH10, TOLLIP, HNRNPA-1, PRKCZ*, and *GLTSCR1* was significantly associated with AUD status (Supplementary Table S11).

### Expression analysis of GAS5 in human postmortem brain

**Fig. 5:**
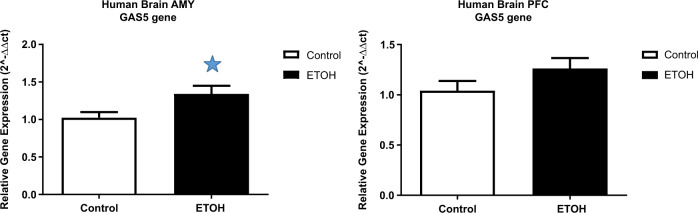
GAS5 expression in human postmortem brain. **a** mRNA expression in human amygdala. **b** mRNA expression in human PFC. Controls *n* = 13, case *n* = 11. *p < 0.05. AMY = amygdala, PFC = prefrontal cortex.

Given the consistent association of *GAS5* methylation with AUD phenotypes, we analyzed expression of *GAS5* in 11 individuals with AUD and 13 controls using human postmortem brain tissues including amygdala and prefrontal cortex. Results showed statistically significantly increased *GAS5* expression in the amygdala in AUD (*p* < 0.05) (Fig. 5), indicating a potential effect of alcohol on *GAS5* expression.

## Discussion

This work represents the largest and most comprehensive EWAS on AUD to date. The large number of significant probes identified in our study may derive both from the statistical power inherent in large cohorts as well as widespread epigenetic changes induced by chronic alcohol exposure. In our discovery cohort, we identified over 5000 DNA methylation associations with AUD after correction for multiple testing. Importantly, while many of these associations are likely biologically relevant, we sought a means beyond statistical stratification to identify the strongest findings from this set. Cross referencing discovery findings with those derived from independent replication sets represents a powerful tool to identify robust findings with likely biological meaning. Using this method, we identified 96 loci consistently associated with both our initially discovered ~5000 FDR significant probes and the 203 FDR significant probes in our first replication set. The overlap between these cohorts was much higher than expected by chance as was an enrichment of multiple biological pathways exhibiting epigenetic change. We further attempted to replicate our findings in a second and third independent population-based cohort. Assessment of the GS cohort revealed 70 of the 96 probes to be significantly associated with alcohol use. The GTP data was generated on an earlier technology, the HM450 microarray, and contained only 35 of the 96 loci above, of which 15 loci were identified to be associated with SCID based AUD diagnoses. Genes associated with these probes included *GAS5, MYST3, UBA3, HECW2, MYH10, RASGRP1, PRKCZ*, and *FKBP5*. Gene ontology (GO) analysis demonstrated strong evidence for AUD associated epigenetic alterations in genes associated with immune response, glucocorticoid signaling and various inflammatory cytokines (Supplementary Table S12). Notably, the effects of alcohol exposure on inflammatory processes are well documented. Alcohol exposure can activate both acute and chronic inflammatory processes, both in brain [50] and liver [51].

Given that methylomic variation across the genome is not independent and likely coregulated by various systematic influences, we sought to further disentangle and model the biological complexity of identified CpG sites in our sample. We used WGCNA to generate a data driven grouping of loci most likely to be coregulated in association with AUD. One module consisting of 50 loci was associated with AUD status and contained a number of loci relevant to alcohol exposure (Result S1), including glucocorticoid signaling and inflammation. Interestingly, our data are in line and partially replicate a recent study of DNA methylation in human postmortem brain, which identified probes in the glucocorticoid receptor and FKBP5 in AUD [22].

Based on these promising findings, both from a single CpG standpoint (robust findings for GAS5 across all datasets) and the WGCNA analyses, we conducted several follow up experiments to investigate biological function with relevance to human stress responsiveness.

First, we established that the AUD WGCNA module was associated with morning cortisol levels. Remarkably, of those 50 loci interrogated, a network mediation analysis suggested that a probe in *GAS5* exhibited the highest evidence for connectedness in association to cortisol regulation by mediating the association with cortisol across a large number of module genes. *GAS5* interacts with the glucocorticoid receptor (GR) to inhibit its transcriptional function [52] by folding into a soluble glucocorticoid response element-like sequence on the GR to mimic GR binding [53]. Furthermore *GAS5* expression may also alter corticotrophin releasing hormone receptor 1 (*CRHR1*) expression [54]. This finding is intriguing, given robust literature supporting an involvement of the corticotropin releasing factor and its receptor in AUD [55–60].

Second, we used neuroimaging paradigms to probe key regions implicated in stress responsiveness in human, to further explore biological mechanisms of identified CpG-related networks. Main brain regions implicated in the human stress response include the amygdala, frontal cortex, and hippocampus, among others [61, 62]. Importantly, we aimed to explore the neuronal and functional relevance of peripherally identified epigenetic changes. As demonstrated by our group previously, such peripherally identified epigenetic effects may have brain relevance when they derive from a systemic factor such as circulating steroid hormones [63] or alcohol [27]. We focused mainly on the frontal cortex, hippocampus and amygdala. To probe the amygdala specifically, we used a fear conditioning and extinction paradigm in individuals with AUD and controls. Our data showed robust association of AUD-associated DNA methylation with amygdala activation to the anticipation of electric shocks in humans. These data suggest that the AUD-associated coregulated module genes are associated with BOLD signals in distinct brain regions, which in turn may reflect brain-related phenotypes (Supplementary Table S10, Result S1). Remarkably, AUD module epigenetic variation was also associated with hippocampal volume, suggesting a further link between peripheral DNA methylation variation and brain phenotypes. Summarizing our endophenotypes studies, we observed that *GAS5* DNA methylation was associated with alcohol use across all studied cohorts both in the periphery and in the brain. Furthermore, we studied *GAS5* gene expression by conducting postmortem studies of AUD patients and controls and found higher *GAS5* expression in the amygdala. This is intriguing, given the fMRI finding in the amygdala, it suggests a potential direct role of GAS5 in this brain region, while further corroborating the functional relevance of our results. The importance of *GAS5* on phenotypic outcomes may be attributed to its implicated role as a ‘hub gene’ regulating glucocorticoid signaling. *GAS5* exhibited strong module membership (hub status) and demonstrated evidence for a high degree of network connectedness in association with cortisol levels, suggesting it may play a central role in the observed association between AUD co-regulated module variation and cortisol status. It further suggests that BOLD-associated epigenetic variation may be a downstream consequence of or interact with dysregulated glucocorticoid signaling. These results are consistent with the known function of *GAS5* and other module genes exhibiting evidence for coregulation such as *FKBP5* as both are involved in modulating glucocorticoid signaling, and broadly, inflammation. As such, previous evidence linking *FKBP5* to AUD may be in some way be related to *GAS5* and should be investigated further. For example, *FKBP5* is transcribed by the GR and, once expressed, represents a potent intracellular inhibitor of GR function [52]. *FKBP5* gene expression has been suggested as an important mediator of the pathways to the development of drinking behavior. Previous studies show that genetic variants within *FKBP5* that affect its expression mediate effects of both poor parental relationships [64] and metacognitions about alcohol on problematic drinking behavior [65]. These findings hint at genetic effects that modulate *FKBP5* expression, which may contribute to AUD. Similar to our results, a recent study showed methylation changes in *FKBP5* in the prefrontal cortices of adults with alcohol use problems [22]. Concurrently, animal studies demonstrate that *FKBP5* expression in mesocorticolimbic dopaminergic neurons mediates the effects of early life stress on alcoholic behavior [64]. While we observed epigenetic associations with *FKBP5* in three cohorts (discovery and replication AUD, GTP), we could not detect such an effect in the GS sample, possibly due to variation in the degree of trauma/early life stress exposure. Moreover, epigenetic variation in *FKBP5* is particularly interesting in the context of fear conditioning, as was tested in our study participants. *FKBP5* may confer risk to fear extinction deficits [66], while, epigenetic regulation of *FKBP5* has been proposed as a mechanism to mediate glucocorticoid exposure enhanced fear extinction in rodents [67].

Despite our promising findings, there are some limitations that should be carefully considered. While we used several cohorts to replicate methylomic variation associated with AUD, it is important to keep in mind that other factors besides alcohol might influence epigenetic signatures, such as clinical heterogeneity and environmental factors including life-experiences, trauma, diet, exercise patterns, and underlying genetic architecture. In addition, the mechanisms by which alcohol leads to widespread epigenetic reprogramming are not addressed by our study. One possibility is that epigenetic change is the downstream consequence of the secondary effects of alcohol exposure such as inflammatory changes or disrupted glucocorticoid signaling. Alternatively, other loci may represent more direct mediators of the effects of alcohol on phenotype or etiological factors leading to the AUD phenotype. For example, acute alcohol consumption can lead to brain expression changes in *PRKCZ* (one of the replicating genes identified in our study that was not a member of the AUD associated coregulated module), suggesting it may be a consequence of alcohol exposure. Alternatively, deletion of *PRKCZ* in mice leads to increased consumption of alcohol [68] suggesting that variation in *PRKCZ* precedes the AUD phenotype. Similarly, methylation at *GAS5* could reflect a mediating effect of alcohol. Previous work has demonstrated that *GAS5* is bound by the catalytic *EZH2* subdomain of polycomb group complex 2 (*PRC2*) [69]. Further, a recent report highlighted a mechanism whereby alcohol exposure resulted in histone 3 lysine 27 trimethylation marks (within the amygdala) on a brain-derived neurotrophic factor associated long non-coding RNA by *EZH2* [70]. Although the degree to which such a mechanism is generalizable for other non-coding RNAs like *GAS5* has yet to be determined, it is possible that DNA methylation of *GAS5* is affected by interaction with PRC2 and which reflects a mediating effect of alcohol via EZH2. As such, the translation from histone modifications to DNA methylation through sequestration of DNA methyltransferase activity at this specific locus will need to be determined through future studies. Importantly, understanding the cause vs. effect nature of the identified associations is not possible in a cross-sectional design such as the one applied in our study and calls for comprehensive longitudinal studies paired with preclinical animal work to better understand etiological mechanisms in humans. Despite this, consistent AUD associated findings across multiple cohorts suggest the possibility of identifying a combined ‘biosignature’ of AUD. A better understanding of the association of epigenetic variation with not only the diagnostic criteria itself, but also disease-associated endophenotypes such as brain imaging alterations may eventually aid in the development of objective clinical tools to assess for exposure to alcohol and the progression through various phases of drinking behavior severity.

A strength of this study is the use of both diagnostic and dimensional definitions of chronic alcohol use. Across cohorts with various definitions, we continued to find the same probes to be differentially methylated in response to alcohol use, suggesting that the probes identified here are particularly robust indicators of alcohol induced epigenetic changes.

## Supplementary information

Supplementary Material
