# Supplementary material for: Epigenome-wide association study and multi-tissue replication of individuals with alcohol use disorder: evidence for abnormal glucocorticoid signaling pathway gene regulation
Source: Mol Psychiatry. 2020 May 12;26(6):2224–37. doi: 10.1038/s41380-020-0734-4 (PMC7658001; doi:10.1038/s41380-020-0734-4)
Supplement: Supplementary file 1 — Supplementary Material [file 41380_2020_734_MOESM1_ESM.pdf]

Supplementary Information

Epigenome-wide association study and multi-tissue replication of individuals with alcohol use disorder: evidence for abnormal glucocorticoid signaling pathway gene regulation

Falk W. Lohoff[1#], Arunima Roy[2], Jeesun Jung[1], Martha Longley[1], Daniel B. Rosoff[1], Audrey Luo[1], Emma O'Connell[1], Jill L. Sorcher[1], Hui Sun[3], Melanie Schwandt[3], Colin A. Hodgkinson[4], David Goldman[4], Reza Momenan[5], Andrew M. McIntosh[6], Mark J. Adams[6], Rosie M. Walker[7,8], Kathryn L. Evans[7,8], David Porteous[7], Alicia K. Smith[9,10], Jisoo Lee[1], Christine Muench[1], Katrin Charlet[1], Toni-Kim Clarke[6], Zachary A. Kaminsky[2]

[1]Section on Clinical Genomics and Experimental Therapeutics, National Institute on Alcohol Abuse and Alcoholism, National Institutes of Health, Bethesda, MD
[2]Royal's Institute of Mental Health Research, University of Ottawa, Ottawa, Canada
[3]Office of the Clinical Director, National Institute on Alcohol Abuse and Alcoholism, National Institutes of Health, Bethesda, MD
[4]Laboratory of Neurogenetics, National Institute on Alcohol Abuse and Alcoholism, National Institutes of Health, Bethesda, MD
[5]Clinical Neuroimaging Research Core, National Institute on Alcohol Abuse and Alcoholism, National Institutes of Health, Bethesda, MD
[6]Division of Psychiatry, Centre for Clinical Brain Sciences, University of Edinburgh, Edinburgh, UK
[7]Medical Genetic Section, Centre for Genomic and Experimental Medicine, Medical Research Council Institute of Genetics and Molecular Medicine, University of Edinburgh, Edinburgh, UK
[8] Centre for Cognitive Ageing and Cognitive Epidemiology, University of Edinburgh, Edinburgh, UK
[9] Department of Gynecology and Obstetrics, Emory University, Atlanta, Georgia, USA
[10]Department of Psychiatry & Behavioral Sciences, Emory University, Atlanta, GA, USA

RESULT S1

A number of loci were associated with BOLD responses in distinct brain regions (Table S8). Among these, *MYH10, RASGRP1, FKBP5, HECW2,* and *PRCKZ* were also significantly replicated as associated with AUD in the GTP cohort, suggesting these may represent some of our most robust findings. Gene expression relevant epigenetic variation in each of these genes may have a plausible mechanism to affect brain-related phenotypes. For example, *RASGRP1* is expressed in the hippocampus and is involved in learning and memory formation [1, 2]. In animals, *RASGRP1*, seems to modulate striatal activity [3]. Epigenetic changes, in response to chronic alcohol use, altering *RASGRP1* expression could conceivably affect fear learning and fear responses via its effects on the hippocampus. Similarly, *MYH10* regulates synaptic plasticity, hippocampal development, memory formation [4, 5], and therefore epigenetic alterations in this gene may affect fear learning. Epigenetic regulation of *HECW2* has been previously shown to be altered in peripheral cells of individuals with chronic alcohol use [6]. The protein encoded by *PRKCZ* is important for neuronal axon specification [7] and long-term potentiation [8]. Alcohol-induced epigenetic variation may therefore lead to differences in brain connectivity and may represent a mechanism explaining our association of *PRKCZ* DNA methylation and prefrontal BOLD signal. Notably, epigenetic variation in *PRKCZ* was replicated in two independent brain tissue samples, and variation in *MYH10* was replicated in one brain tissue sample, further suggesting that these two findings may be brain relevant. We also identified the *AHRR* gene as being associated with neural functioning during a fear conditioning task. *AHRR* has been identified in previous studies as a biomarker for smoking behavior [9]. Smoking and nicotine use is often comorbid with alcohol, which may explain the association of *AHRR* with BOLD signals in our sample of participants with AUD. Despite this, *AHRR* has gained recent interest in the study of posttraumatic stress disorder (PTSD), a disorder believed to be related to dysregulation of the fear circuitry and where comorbid AUD status worsens treatment outcomes [10, 11]. Other genes identified include *AMBRA1*, well known for its role in neurodevelopmental disorders [12] and recently shown to be involved in maintaining a hippocampal inhibitory/excitatory balance [13], *NFAT*, which is involved in neural growth [14], and *RARA. RARA,* or retinoic acid receptor alpha, modulates neurogenesis and has been implicated in a variety of neural abnormalities, including amyloid plaque formation [15], alterations in neural growth and differentiation in response to stress [16] and regulation of corticotropin releasing hormone (CRH) expression in neural tissue [17]. Importantly, ethanol exposure was demonstrated to impair expression of retinoic acid receptors in cerebellar neurons of rodents [18].

Other genes among those 96 loci consistently overlapped between our discovery and replication cohorts may be of interest. For example, an association with the *TOLLIP* gene may be important for mediating the effects of alcohol on downstream inflammatory processes. Previous animal studies confirm our observations in humans, as elevated DNA methylation in the *TOLLIP* gene was identified in livers of rats with high ethanol diets or with high as compared to low blood alcohol levels [19]. *TOLLIP* plays an important role in mediating Toll like receptor 4-mediated neuroinflammation in the brain [20] and has been implicated as playing an important role in inflammatory and infectious diseases [21].

Table S1. Sample demographics and characteristics for the discovery cohort (blood, N=539)

	Controls (n = 203)		Participants with AUD (n = 336)		
	Mean	SD	Mean	SD	p value
Age	34.64	12.54	44.29	10.92	≤0.001
Fagerström Test of Nicotine Dependence scores	0.14	0.85	2.32	2.59	≤0.001
Number of Heavy Drinking Days in Past 90 Days	4.18	12.93	63.65	27.30	≤0.001
Average Number of Drinks per Drinking Day	2.39	1.65	12.38	7.86	≤0.001
Total AUDIT scores	3.69	3.52	23.44	8.59	≤0.001
Baseline Anxiety Scores	0.96	2.08	10.50	7.55	≤0.001
Spielberger State-Trait Anxiety Inventory Score	28.29	6.80	46.43	12.42	≤0.001
Perceived Stress Scale Total Score	8.46	5.35	19.78	7.87	≤0.001
Alcohol Dependence Scale Scores	0.87	1.85	19.85	8.40	≤0.001
Stressful Life Events - Total Scores	2.92	2.92	2.64	2.79	0.315
	n	%	n	%	p value
Gender					
Females	102	50.24	136	40.59	0.067
Males	101	49.75	199	59.40	
Race					0.075
Black/African American	82	40.39	167	49.85	
White	121	59.60	168	50.14	
Ethnicity					≤0.001
Hispanic or Latino	13	6.40	2	0.59	
Not Hispanic or Latino	190	93.59	333	99.40	
Generalized Anxiety Disorder					≤0.001
Yes	1	0.50	46	13.77	
No	199	99.5	288	86.22	
Depressive disorder - not otherwise specified					1.00
Yes	0	0.00	1	0.29	
No	200	100.00	333	99.70	

Table S2. Sample demographics and characteristics for the first replication cohort (blood, N=86)

	Healthy Controls (N = 43)	Individuals with Alcohol Dependence (N = 43)	P-Value
Gender, N, (%)			
Male	19 (44.19)	29 (67.44)	**.030**
Female	24 (55.81)	14 (32.56)	
Age, mean years (SD)	38.85 (11.78)	45.95 (11.07)	**.005**
FTND, mean (SD)	0 (0.0)	1.93 (2.49)	**<.0001**
History of Early Life Stress, N (%)	17 (39.53)	26 (60.47)	**.052**
Number of Heavy Drinking Days in Past 90 Days, mean (SD)	0.47 (1.82)	67.63 (27.81)	**<.0001**
Average Number of Drinks per Drinking Day, mean (SD)	1.35 (0.89)	14.34 (8.97)	**<.0001**
Montgomery-Asberg Depression Score, mean (SD)	1.26 (2.84)	13.98 (9.53)	**<.0001**
BSA Anxiety, mean (SD)	0.81 (1.58)	10.53 (7.84)	**<.0001**
STAI Score, mean (SD)	28.23 (7.74)	47.98 (11.32)	**<.0001**
PSS Stress Scale, mean (SD)	9.62 (6.47)	21.44 (7.04)	**<.0001**
ADS, mean (SD)	0.71 (1.50)	21.33 (8.52)	**<.0001**
CIWA, mean (SD)	0.12 (0.39)	4.56 (3.77)	**<.0001**
DSM-IV/DSM-5 Diagnosis, Lifetime			
Posttraumatic Stress Disorder	1 (2.33)	5 (11.63)	.090
Other Specified Trauma- and Stressor-Related Disorder	0 (0.0)	1 (2.33)	.314
Generalized Anxiety Disorder	0 (0.0)	4 (9.30)	**.041**
Anxiety Disorder Not Otherwise Specified/Other Specified Anxiety Disorder	0 (0.0)	5 (11.63)	**.021**
Recurrent Major Depressive Disorder/Major Depressive Disorder	1 (2.33)	11 (25.58)	**.002**
Dysthymic Disorder/Persistent Depressive Disorder	1 (2.33)	4 (9.30)	.167
Other Specified Depressive Disorder	1 (2.33)	1 (2.33)	1.0

Note. SD = standard deviation, FTND = Fagerström Test of Nicotine Dependence, BSA = Brief Scale for Anxiety, STAI = Spielberger State-Trait Anxiety Inventory-Y2, PSS = Perceived Stress Scale, ADS = Alcohol Dependence Scale, CIWA = Clinical Withdrawal Assessment-Alcohol Revised. DSM-IV comorbidities include current and past diagnoses; DSM-5 comorbidities include lifetime diagnoses. Boldface indicates significant between-group differences.

Table S3. Overlap of probes from discovery and replication datasets showing significant associations of alcohol use disorder with DNA methylation

Probe	Gene	Discovery cohort (n = 539)			First blood replication cohort (n = 86)		
		B	SE	Adjusted p	B	SE	Adjusted p
cg00238470	*TRAPPC3*	-0.009	0.002	0.03231	-0.027	0.006	0.041407
cg00475490	*PRSS23*	-0.017	0.003	0.00092	-0.042	0.008	0.031448
cg00501876	*CSRNP1*	-0.019	0.003	6.26E-07	-0.029	0.006	0.035794
cg00505318	*UBA3*	-0.012	0.002	0.000369	-0.026	0.005	0.030222
cg00666295	*SNHG1*	-0.017	0.004	0.00152	-0.043	0.009	0.038455
cg01940273		-0.056	0.006	1.05E-13	-0.078	0.013	0.002796
cg02517348		-0.026	0.004	8E-07	-0.055	0.009	0.005858
cg02583484	*HNRNPA1*	-0.030	0.003	3.66E-13	-0.048	0.008	0.009252
cg02606535	*TSC2*	0.026	0.003	4.22E-10	0.039	0.007	0.012292
cg03401021	*LINC01599*	-0.007	0.002	0.037015	-0.020	0.004	0.047199
cg03603381	*RASGRP1*	-0.015	0.003	0.000519	-0.038	0.008	0.038455
cg03883939	*AMIGO3, RNF123*	0.014	0.003	0.00488	0.039	0.007	0.009145
cg04406114	*POU2F2*	0.010	0.002	0.003367	0.024	0.005	0.03414
cg05299572	*BNIPL*	-0.010	0.002	0.010466	-0.032	0.006	0.008912
cg05575921	*AHRR*	-0.104	0.011	1.57E-13	-0.144	0.024	0.003664
cg05994753	*LOC101927588*	-0.011	0.003	0.030663	-0.048	0.007	0.002492
cg06466940	*ARRB1*	-0.010	0.002	0.00203	-0.027	0.006	0.032756
cg06644515	*GAS5*	-0.015	0.003	0.000536	-0.044	0.007	0.002796
cg07267541	*LURAP1L*	-0.028	0.005	1.77E-05	-0.059	0.012	0.034704
cg07687574	*PIK3R5*	-0.015	0.003	0.000463	-0.035	0.007	0.031448
cg08109625	*PRKCZ*	0.011	0.003	0.043633	0.036	0.007	0.02318
cg08230167	*LURAP1L*	-0.034	0.004	1.94E-09	-0.055	0.011	0.030786

Probe	Gene	Discovery cohort (n = 539)			First blood replication cohort (n = 86)		
		**B**	**SE**	**Adjusted p**	**B**	**SE**	**Adjusted p**
cg08311647	*USP6NL*	-0.013	0.003	0.008676	-0.036	0.006	0.008912
cg08832418	*NOP56*	-0.018	0.004	0.000776	-0.048	0.009	0.012889
cg09095403	*HPS4*	0.010	0.002	0.006185	0.024	0.005	0.038455
cg09511513	*ATP6V1B2*	-0.036	0.005	3.4E-08	-0.071	0.012	0.003664
cg09881793		-0.010	0.002	0.006197	-0.023	0.004	0.023067
cg09975715	*MYH10*	0.019	0.003	3.75E-05	0.042	0.008	0.02848
cg10204912	*NFATC2*	-0.013	0.003	0.006562	-0.035	0.007	0.022368
cg10558233		-0.026	0.005	0.000898	-0.066	0.013	0.031448
cg11095027	*TOLLIP*	0.024	0.003	6.58E-09	0.033	0.007	0.032658
cg11589723	*GLTSCR1*	0.011	0.002	0.000655	0.031	0.006	0.045373
cg12169251	*MYH9*	-0.010	0.003	0.037929	-0.028	0.006	0.036825
cg12464134		-0.013	0.003	0.027829	-0.036	0.007	0.030222
cg12726743	*CAMK2G*	-0.010	0.003	0.033766	-0.033	0.007	0.041959
cg12792732	*ATP6V1H*	-0.018	0.004	0.002848	-0.050	0.009	0.010326
cg13683827	*FAM113B*	-0.010	0.002	0.005374	-0.032	0.006	0.012228
cg14222701	*AMBRA1*	0.011	0.002	0.002041	0.033	0.006	0.005663
cg14316231	*MYST3*	-0.012	0.003	0.027805	-0.040	0.008	0.037808
cg14334350	*NOTCH1*	0.017	0.003	0.000716	0.044	0.008	0.008912
cg14377032	*HTRA2*	-0.016	0.004	0.005202	-0.038	0.008	0.031039
cg14391737	*PRSS23*	-0.034	0.005	3.04E-06	-0.059	0.012	0.039326
cg15112362	*ZFP36L1*	-0.007	0.001	9.34E-05	-0.012	0.002	0.016451
cg15130459	*RPL37*	-0.016	0.004	0.039986	-0.044	0.009	0.032658
cg15251675		-0.020	0.004	0.000945	-0.057	0.009	0.004994
cg15356233		-0.020	0.004	0.002329	-0.054	0.011	0.042382

Probe	Gene	Discovery cohort (n = 539)			First blood replication cohort (n = 86)		
		B	SE	Adjusted p	B	SE	Adjusted p
cg15408734		-0.008	0.001	0.000647	-0.020	0.004	0.014113
cg15554126	*PEX14*	0.011	0.003	0.041142	0.036	0.006	0.008912
cg15627993	*LDLRAD4*	0.034	0.005	8.83E-09	0.059	0.012	0.028068
cg15705813		-0.021	0.003	1.03E-07	-0.038	0.006	0.003906
cg16290996	*GAS5*	-0.014	0.003	0.003935	-0.043	0.008	0.008912
cg16562703	*DST*	-0.011	0.003	0.008353	-0.031	0.006	0.028068
cg16734637	*FOXP1*	-0.023	0.003	3.88E-08	-0.048	0.009	0.020263
cg16825803		-0.013	0.004	0.049811	-0.040	0.008	0.03414
cg17679916		-0.017	0.004	0.015841	-0.057	0.010	0.011075
cg17739917	*RARA*	-0.047	0.005	4.23E-12	-0.069	0.011	0.002492
cg17825846		-0.017	0.003	0.000393	-0.038	0.007	0.02318
cg18159646	*SNORD30, SNHG1*	-0.008	0.002	0.03442	-0.029	0.005	0.009934
cg18268027	*ITPR1*	-0.021	0.003	1.59E-07	-0.045	0.009	0.020263
cg18387338		-0.032	0.004	1.5E-10	-0.046	0.009	0.028068
cg18581607	*PLD2*	0.036	0.006	2.11E-06	0.071	0.014	0.028068
cg18696027	*B2M*	-0.009	0.002	0.010624	-0.027	0.005	0.008912
cg18752527	*HECW2*	-0.007	0.001	0.001132	-0.017	0.003	0.019583
cg18917643		-0.027	0.004	6.62E-09	-0.057	0.010	0.009844
cg19398525	*ZNRF2*	-0.013	0.003	0.002367	-0.038	0.007	0.012889
cg19918116	*DNAH10*	-0.015	0.003	0.00017	-0.031	0.006	0.030222
cg20005742		0.020	0.005	0.013978	0.064	0.013	0.038455
cg20184330	*ELMSAN1*	-0.022	0.003	2.37E-07	-0.038	0.008	0.037172
cg20540629	*SKAP1*	-0.009	0.002	0.003045	-0.022	0.005	0.036726

Probe	Gene	Discovery cohort (n = 539)			First blood replication cohort (n = 86)		
		B	SE	Adjusted p	B	SE	Adjusted p
cg20625334	*STAM2*	-0.026	0.004	1.39E-06	-0.061	0.012	0.023067
cg20725067	*UBE2E1*	-0.016	0.003	0.000277	-0.039	0.008	0.02318
cg20732869	*SLC1A2*	-0.020	0.003	3.13E-06	-0.050	0.009	0.011735
cg20813374	*FKBP5*	-0.020	0.004	0.000139	-0.045	0.009	0.030222
cg21021065		-0.013	0.003	0.002178	-0.036	0.007	0.028239
cg21053786		-0.020	0.003	6.04E-06	-0.044	0.009	0.030949
cg21095779	*IL1RAP*	-0.029	0.005	0.00019	-0.077	0.014	0.009145
cg21108805	*WIF1*	-0.026	0.004	1.35E-06	-0.056	0.010	0.008912
cg21566642		-0.072	0.008	1.57E-13	-0.112	0.017	0.002526
cg22140810	*FAM131B*	0.011	0.002	0.001554	0.027	0.006	0.032658
cg22158648	*CABLES1*	-0.012	0.003	0.00906	-0.035	0.007	0.030222
cg22818074	*MAP2K1*	0.029	0.006	0.0028	0.086	0.017	0.030222
cg22998476		-0.013	0.003	0.004702	-0.043	0.007	0.00512
cg23010288		-0.014	0.003	0.014928	-0.046	0.009	0.012889
cg23098235	*HOXB3*	-0.010	0.002	0.00101	-0.022	0.004	0.032658
cg23999572	*LINC-PINT*	-0.025	0.004	2.9E-05	-0.053	0.010	0.012292
cg24797066		-0.012	0.002	5.99E-05	-0.025	0.005	0.031039
cg25001882		-0.013	0.003	0.002943	-0.033	0.006	0.010799
cg25436965	*TMEM72-AS1*	-0.014	0.003	0.007391	-0.044	0.008	0.012651
cg25537245		-0.006	0.001	0.010192	-0.016	0.003	0.049458
cg25615944	*RPL23*	-0.011	0.003	0.028784	-0.035	0.006	0.012228
cg26695233	*LINC01126*	-0.015	0.003	0.000686	-0.042	0.008	0.010326
cg26952862		-0.014	0.002	9.62E-06	-0.029	0.006	0.030222
cg27372654	*MEF2C*	-0.023	0.003	4.18E-11	-0.038	0.008	0.038214

Probe	Gene	Discovery cohort (n = 539)			First blood replication cohort (n = 86)		
		**B**	**SE**	**Adjusted p**	**B**	**SE**	**Adjusted p**
cg27412523	*UTRN*	-0.011	0.003	0.027037	-0.021	0.004	0.039067
cg27537125		-0.013	0.002	2.42E-07	-0.024	0.004	0.012292
cg27650500		-0.008	0.002	0.042548	-0.032	0.006	0.009782

Table S4. Associations between methylation at the 96 target probes with alcohol use in the Scottish Family Health Study listed in descending order of significance

		Second blood replication cohort (GS) (n = 4301)		
**Probes**	**Gene**	**B**	**SE**	**p**
cg06644515	*GAS5*	43.260	0.002	3.07E-24
cg15705813		17.259	0.002	8.53E-13
cg18159646	*SNORD30, SNHG1*	16.008	0.002	3.05E-12
cg18581607	*PLD2*	15.225	0.002	6.79E-12
cg26952862		14.884	0.003	9.61E-12
cg25436965	*TMEM72-AS1*	14.765	0.002	1.09E-11
cg02583484	*HNRNPA1*	14.749	0.002	1.1E-11
cg18917643		13.691	0.002	3.25E-11
cg14222701	*AMBRA1*	12.809	0.002	8.02E-11
cg02606535	*TSC2*	12.019	0.002	1.8E-10
cg07267541	*LURAP1L*	7.115	0.002	2.77E-08
cg16290996	*GAS5*	7.078	0.003	2.88E-08
cg11095027	*TOLLIP*	7.009	0.001	3.09E-08
cg09095403	*HPS4*	6.940	0.002	3.32E-08
cg15112362	*ZFP36L1*	6.263	0.002	6.66E-08
cg14334350	*NOTCH1*	4.796	0.002	3.04E-07
cg15251675		4.413	0.002	4.52E-07
cg20625334	*STAM2*	3.884	0.002	7.83E-07
cg05299572	*BNIPL*	3.447	0.002	1.23E-06
cg08832418	*NOP56*	2.909	0.002	2.16E-06
cg16562703	*DST*	2.778	0.003	2.47E-06
cg00666295	*SNHG1*	1.821	0.003	6.71E-06
cg03883939	*AMIGO3, RNF123*	1.766	0.002	7.11E-06
cg11589723	*GLTSCR1*	1.627	0.003	8.22E-06
cg20725067	*UBE2E1*	1.260	0.002	1.21E-05
cg13683827	*FAM113B*	1.152	0.002	1.35E-05
cg15554126	*PEX14*	1.127	0.002	1.39E-05
cg05994753	*LOC101927588*	0.957	0.001	1.66E-05
cg09511513	*ATP6V1B2*	0.846	0.003	1.86E-05
cg01940273		0.811	0.003	1.93E-05
cg02517348		0.805	0.003	1.95E-05
cg21021065		0.772	0.002	2.02E-05
cg21566642		0.507	0.003	2.66E-05
cg21053786		0.184	0.002	3.74E-05
cg27650500		0.105	0.001	4.06E-05
cg17739917	*RARA*	-0.041	0.003	4.74E-05
cg20732869	*SLC1A2*	-0.638	0.003	8.92E-05
cg20005742		-0.766	0.003	0.000102
cg03603381	*RASGRP1*	-0.827	0.001	0.000109
cg25001882		-0.894	0.002	0.000117
cg08230167	*LURAP1L*	-1.564	0.003	0.000238
cg12792732	*ATP6V1H*	-1.732	0.002	0.000285
cg07687574	*PIK3R5*	-1.743	0.002	0.000288
cg26695233	*LINC01126*	-2.173	0.002	0.000457
cg22998476		-2.227	0.002	0.000484

Probes	Gene	Second blood replication cohort (GS) (n = 4301) B	SE	p
cg04406114	*POU2F2*	-2.318	0.002	0.000534
cg05575921	*AHRR*	-2.406	0.005	0.000587
cg23999572	*LINC-PINT*	-2.686	0.003	0.000793
cg27372654	*MEF2C*	-2.872	0.002	0.00097
cg00505318	*UBA3*	-3.693	0.002	0.002363
cg18696027	*B2M*	-4.371	0.002	0.004982
cg25537245		-4.373	0.002	0.004994
cg15408734		-4.571	0.002	0.006216
cg15130459	*RPL37*	-4.785	0.002	0.007891
cg15356233		-4.792	0.003	0.007956
cg09881793		-4.856	0.001	0.00854
cg22818074	*MAP2K1*	-4.861	0.003	0.008595
cg10558233		-4.953	0.003	0.009519
cg20184330	*ELMSAN1*	-4.979	0.002	0.009807
cg21108805	*WIF1*	-5.020	0.003	0.010271
cg27412523	*UTRN*	-5.202	0.002	0.012601
cg09975715	*MYH10*	-5.262	0.002	0.013482
cg17825846		-5.631	0.002	0.020533
cg14316231	*MYST3*	-5.724	0.002	0.022848
cg23010288		-6.092	0.003	0.035043
cg18387338		-6.240	0.003	0.041703
cg15627993	*LDLRAD4*	-6.357	0.002	0.047886
cg00238470	*TRAPPC3*	-6.394	0.002	0.050039
cg18752527	*HECW2*	-6.599	0.003	0.064027
cg03401021	*LINC01599*	-6.663	0.002	0.069262
cg14391737	*PRSS23*	-6.783	0.004	0.08024
cg10204912	*NFATC2*	-6.942	0.002	0.097679
cg08311647	*USP6NL*	-7.041	0.002	0.110686
cg18268027	*ITPR1*	-7.048	0.002	0.111694
cg14377032	*HTRA2*	-7.076	0.002	0.115626
cg20813374	*FKBP5*	-7.107	0.002	0.120325
cg00475490	*PRSS23*	-7.296	0.003	0.153708
cg06466940	*ARRB1*	-7.357	0.002	0.166567
cg17679916		-7.477	0.003	0.195905
cg22158648	*CABLES1*	-7.488	0.002	0.198911
cg20540629	*SKAP1*	-7.673	0.002	0.257843
cg08109625	*PRKCZ*	-7.785	0.002	0.303896
cg23098235	*HOXB3*	-7.792	0.001	0.30712
cg16734637	*FOXP1*	-7.824	0.002	0.322647
cg00501876	*CSRNP1*	-7.843	0.001	0.332356
cg12169251	*MYH9*	-7.856	0.002	0.338724
cg12726743	*CAMK2G*	-7.875	0.002	0.349013
cg16825803		-8.039	0.002	0.458935
cg19398525	*ZNRF2*	-8.079	0.003	0.493787
cg12464134		-8.208	0.002	0.645723
cg25615944	*RPL23*	-8.253	0.002	0.729583
cg24797066		-8.307	0.001	0.912369

Probes	Gene	Second blood replication cohort (GS) (n = 4301)		
		B	SE	p
cg19918116	*DNAH10*	-8.312	0.002	0.961153
cg21095779	*IL1RAP*	#N/A	#N/A	#N/A
cg22140810	*FAM131B*	#N/A	#N/A	#N/A
cg27537125		#N/A	#N/A	#N/A

Table S5. Probes from the Grady trauma project showing an overlap with sites detected in the discovery and first blood replication cohort listed in descending order of significance

Probe	Gene	Third blood replication cohort (GTP) (n = 391)		
		B	SE	p
cg06644515	*GAS5*	-0.022	0.006	0.000708
cg15130459	*RPL37*	-0.025	0.009	0.004361
cg20813374	*FKBP5*	-0.014	0.005	0.011410
cg01940273		-0.023	0.009	0.011891
cg14316231	*MYST3*	-0.014	0.006	0.017894
cg21566642		-0.028	0.012	0.022707
cg16290996	*GAS5*	-0.018	0.008	0.024696
cg08109625	*PRKCZ*	0.015	0.007	0.026666
cg18917643		-0.013	0.006	0.033940
cg00505318	*UBA3*	-0.018	0.009	0.036821
cg27537125		-0.009	0.004	0.038591
cg10558233		-0.022	0.010	0.038757
cg15705813		-0.014	0.007	0.046109
cg20005742		0.016	0.008	0.049435
cg14334350	*NOTCH1*	0.011	0.006	0.076890
cg00501876	*CSRNP1*	-0.011	0.006	0.083695
cg25537245		-0.005	0.003	0.085426
cg18159646	*SNORD30, SNHG1*	-0.009	0.006	0.128776
cg04406114	*POU2F2*	0.009	0.006	0.161952
cg02583484	*HNRNPA1*	-0.009	0.007	0.191467
cg03603381	*RASGRP1*	-0.007	0.006	0.200415
cg25615944	*RPL23*	-0.008	0.008	0.320031
cg22998476		-0.007	0.007	0.324196
cg15408734		-0.003	0.003	0.326626
cg18752527	*HECW2*	0.004	0.005	0.442937
cg11095027	*TOLLIP*	0.005	0.007	0.448987
cg09975715	*MYH10*	0.002	0.005	0.639299
cg15554126	*PEX14*	-0.002	0.004	0.648408
cg11589723	*GLTSCR1*	-0.001	0.002	0.746305
cg18696027	*B2M*	0.002	0.006	0.791865
cg20625334	*STAM2*	-0.002	0.008	0.795296
cg22158648	*CABLES1*	0.001	0.007	0.881738
cg13683827	*FAM113B*	0.000	0.005	0.935363

Table S6. Associations between probe methylation and alcohol use disorder status adjusted for scores on Fagerstrom Nicotine dependence test, age, sex, race, and cell type

Probes	Gene	B	SE	p
cg02606535	*TSC2*	-0.026	0.004	2.58e-13
cg02583484	*HNRNPA1*	0.026	0.003	2.82e-13
cg27372654	*MEF2C*	0.022	0.003	8.51e-13
cg18917643		0.026	0.004	4.67e-11
cg11095027	*TOLLIP*	-0.021	0.003	4.39e-10
cg15705813		0.020	0.003	8.09e-10
cg08230167	*LURAP1L*	0.028	0.005	2.61e-09
cg16734637	*FOXP1*	0.021	0.003	4.09e-09
cg09511513	*ATP6V1B2*	0.031	0.005	5.88e-09
cg20625334	*STAM2*	0.025	0.004	8.80e-09
cg18387338		0.024	0.004	1.34e-08
cg01940273		0.033	0.006	1.51e-08
cg02517348		0.024	0.004	1.91e-08
cg15627993	*LDLRAD4*	-0.027	0.005	2.99e-08
cg20184330	*ELMSAN1*	0.019	0.003	5.66e-08
cg20732869	*SLC1A2*	0.019	0.003	5.73e-08
cg21566642		0.040	0.007	1.06e-07
cg20725067	*UBE2E1*	0.017	0.003	2.84e-07
cg18268027	*ITPR1*	0.016	0.003	3.31e-07
cg26952862		0.012	0.002	4.71e-07
cg21108805	*WIF1*	0.022	0.004	5.20e-07
cg23999572	*LINC-PINT*	0.023	0.005	5.33e-07
cg17739917	*RARA*	0.026	0.005	6.24e-07
cg18581607	*PLD2*	-0.030	0.006	6.83e-07
cg00505318	*UBA3*	0.012	0.002	1.03e-06
cg06644515	*GAS5*	0.016	0.003	1.51e-06
cg00666295	*SNHG1*	0.018	0.004	1.67e-06
cg21053786		0.017	0.003	2.16e-06
cg11589723	*GLTSCR1*	-0.012	0.002	2.24e-06
cg19918116	*DNAH10*	0.014	0.003	2.26e-06
cg15112362	*ZFP36L1*	0.006	0.001	2.42e-06
cg05575921	*AHRR*	0.046	0.010	3.38e-06
cg09975715	*MYH10*	-0.017	0.004	3.76e-06
cg03603381	*RASGRP1*	0.015	0.003	4.01e-06
cg20005742		-0.024	0.005	4.79e-06
cg04406114	*POU2F2*	-0.010	0.002	7.77e-06
cg27537125		0.009	0.002	8.11e-06
cg26695233	*LINC01126*	0.014	0.003	9.33e-06
cg14334350	*NOTCH1*	-0.016	0.004	1.11e-05
cg07267541	*LURAP1L*	0.022	0.005	1.21e-05
cg20813374	*FKBP5*	0.017	0.004	1.29e-05
cg09095403	*HPS4*	-0.011	0.003	1.40e-05
cg00501876	*CSRNP1*	0.013	0.003	1.56e-05
cg25436965	*TMEM72-AS1*	0.015	0.003	1.58e-05
cg12792732	*ATP6V1H*	0.018	0.004	1.64e-05
cg23098235	*HOXB3*	0.009	0.002	1.65e-05
cg15356233		0.020	0.005	2.08e-05

Probes	Gene	B	SE	p
cg03883939	*AMIGO3;RNF123*	-0.014	0.003	2.55e-05
cg08832418	*SNORA51*	0.016	0.004	3.27e-05
cg07687574	*PIK3R5*	0.013	0.003	3.28e-05
cg19398525	*ZNRF2*	0.012	0.003	3.55e-05
cg17825846		0.014	0.003	4.74e-05
cg15251675		0.017	0.004	5.45e-05
cg15408734		0.006	0.002	6.33e-05
cg14222701	*AMBRA1*	-0.010	0.003	7.19e-05
cg09881793		0.010	0.002	7.74e-05
cg22140810	*FAM131B*	-0.010	0.002	8.55e-05
cg03401021	*LINC01599*	0.007	0.002	9.18e-05
cg18752527	*HECW2*	0.006	0.002	9.35e-05
cg06466940	*ARRB1*	0.009	0.002	0.00010
cg21095779	*IL1RAP*	0.022	0.006	0.00013
cg23010288		0.014	0.004	0.00015
cg27412523	*UTRN*	0.012	0.003	0.00017
cg10558233		0.021	0.006	0.00024
cg18159646	*SNORD30;SNHG1*	0.009	0.002	0.00026
cg05994753	*LOC101927588*	0.011	0.003	0.00026
cg08311647	*USP6NL*	0.012	0.003	0.00030
cg22158648	*CABLES1*	0.011	0.003	0.00038
cg13683827	*FAM113B*	0.009	0.003	0.00040
cg10204912	*NFATC2*	0.011	0.003	0.00048
cg21021065		0.010	0.003	0.00058
cg05299572	*BNIPL*	0.009	0.002	0.00059
cg08109625	*PRKCZ*	-0.011	0.003	0.00062
cg16290996	*GAS5*	0.011	0.003	0.00073
cg18696027	*B2M*	0.007	0.002	0.00117
cg16562703	*DST*	0.009	0.003	0.00135
cg14377032	*HTRA2*	0.012	0.004	0.00140
cg25615944	*RPL23*	0.010	0.003	0.00143
cg16825803		0.012	0.004	0.00163
cg22818074	*MAP2K1*	-0.021	0.007	0.00196
cg12726743	*CAMK2G*	0.008	0.003	0.00261
cg15554126	*PEX14*	-0.009	0.003	0.00269
cg17679916		0.013	0.004	0.00274
cg12464134		0.011	0.004	0.00338
cg12169251	*MYH9*	0.008	0.003	0.00342
cg22998476		0.009	0.003	0.00369
cg15130459	*RPL37*	0.014	0.005	0.00391
cg25537245		0.004	0.002	0.00634
cg24797066		0.006	0.002	0.00830
cg00238470	*TRAPPC3*	0.006	0.002	0.00951
cg14391737	*PRSS23*	0.013	0.005	0.01158
cg14316231	*MYST3*	0.007	0.003	0.02097
cg20540629	*SKAP1*	0.004	0.002	0.02485
cg27650500		0.005	0.002	0.02629
cg00475490	*PRSS23*	0.007	0.003	0.03322
cg25001882		0.006	0.003	0.03976

Table S7. Associations between probe methylation and cumulative alcohol exposure (average number of drinks per day multiplied by the number of years since their self-reported age of drinking inception)

Probes	Gene	B	SE	p
cg01940273		-1208.85	192.14	1.17E-09
cg05575921	AHRR	-635.12	102.95	2.32E-09
cg21566642		-873.3	149.97	1.54E-08
cg18387338		-1594.83	275.68	1.89E-08
cg18581607	PLD2	1080.34	204.97	2.67E-07
cg14391737	PRSS23	-1186.09	227.73	3.63E-07
cg02583484	HNRNPA1	-1769.66	341.94	4.27E-07
cg08230167	LURAP1L-AS1;LURAP1L	-1252.49	243.21	4.84E-07
cg25001882		-2302.73	462.42	1.10E-06
cg21053786		-1630.59	331.19	1.43E-06
cg03603381	RASGRP1	-1943.38	399.95	1.94E-06
cg15627993	LDLRAD4	1088.99	227.87	2.81E-06
cg18917643		-1233.94	263.15	4.24E-06
cg20813374	FKBP5	-1398.09	318.79	1.62E-05
cg22158648	CABLES1	-1266.73	290.29	1.78E-05
cg17825846		-1386.23	320.21	2.07E-05
cg16734637	FOXP1	-1409.21	326.75	2.22E-05
cg09511513	ATP6V1B2	-937.69	218.79	2.49E-05
cg18268027	ITPR1	-1416.55	331.47	2.62E-05
cg11095027	TOLLIP	1408.58	330.11	2.69E-05
cg17739917	RARA	-834.14	196.53	2.96E-05
cg16562703	RNU6-71P;DST	-1823.75	435.11	3.69E-05
cg10558233		-907.77	220.48	5.01E-05
cg25615944	RPL23	-1620.08	393.65	5.05E-05
cg02517348		-1128.25	279.2	6.83E-05
cg22818074	MAP2K1	590.93	146.83	7.30E-05
cg27372654	MEF2C	-1288.2	324.06	8.90E-05
cg03401021	LINC01599	-2181.67	550.53	9.35E-05
cg26695233	LINC01126	-977.87	249.11	0.00011
cg06466940	ARRB1	-1289.38	330.84	0.00012
cg18752527	HECW2	-2480.04	637.29	0.00012
cg20725067	UBE2E1	-1177.97	304.82	0.00014
cg23098235	HOXB3	-1470.94	386.72	0.00017
cg21108805	WIF1	-1058.03	283.1	0.00022
cg02606535	TSC2;TSC2;TSC2	1260	345.3	0.00031
cg20184330	ELMSAN1;ELMSAN1	-1129.01	309.9	0.00032
cg22998476		-1191.32	335.88	0.00045
cg25436965	TMEM72-AS1	-1193.43	338.67	0.00049
cg13683827	FAM113B	-1496.92	436.11	0.00069
cg19398525	ZNRF2	-958.94	280.1	0.00071
cg07267541	LURAP1L;LURAP1L-AS1	-858.14	260.33	0.0011
cg14377032	AUP1;HTRA2	-903.34	275.2	0.0012
cg12464134		-1154.03	358.7	0.0014
cg21021065		-1266.38	398.46	0.0016
cg24797066		-611.36	192.7	0.0017
cg16825803		-783.26	247.31	0.0017

Probes	Gene	B	SE	p
cg14316231	MYST3	-1141.76	368.58	0.0021
cg09975715	MYH10	932.7	304.03	0.0024
cg00475490	PRSS23	-986.04	327.27	0.0028
cg15251675		-806.86	276.73	0.0038
cg10204912	NFATC2	-808.9	292.5	0.0061
cg21095779	IL1RAP	-586.88	217.18	0.0073
cg00501876	CSRNP1	-777.68	305	0.011
cg05994753	LOC101927588	-1030.28	415.03	0.014
cg27650500		-1212.93	499.62	0.016
cg20625334	STAM2	-635.79	269.8	0.019
cg16290996	GAS5b	-874.43	374.13	0.020
cg23010288		-669.24	286.72	0.020
cg00238470	TRAPPC3	-1205.5	525.88	0.023
cg26952862		-1007.06	449.27	0.026
cg20732869	SLC1A2	-668.73	306.87	0.030
cg15356233		-560.27	266.36	0.036
cg27537125		-992.08	483.51	0.041
cg18696027	B2M	-782.82	407.12	0.055
cg05299572	C1orf56;BNIPL	-716.34	423.7	0.092
cg15705813		-426.22	289.75	0.142
cg23999572	LINC-PINT	-309.48	225.37	0.171
cg22140810	FAM131B	672.13	495.18	0.176
cg12792732	ATP6V1H	-343.28	286.96	0.233
cg12726743	CAMK2G	-465.71	391.31	0.235
cg09095403	HPS4	-291.22	245.36	0.236
cg11589723	GLTSCR1	535.92	454.18	0.239
cg15408734		-714.02	667.88	0.286
cg15554126	PEX14	422.95	410.97	0.304
cg14334350	NOTCH1	283.74	279.37	0.311
cg07687574	PIK3R5	-347.32	348.64	0.320
cg25537245		-705.46	713.52	0.324
cg20005742		212.24	230.22	0.357
cg03883939	AMIGO3;RNF123	271.16	306.39	0.377
cg15130459	SNORD72;RPL37	-207.5	252.16	0.411
cg06644515	SNORD47;GAS5a	-196.75	321.08	0.541
cg08832418	SNORA51;NOP56	-179.24	298.28	0.548
cg12169251	MYH9	163.41	335.81	0.627
cg00666295	SNORD22;SNHG1	-130.75	279.65	0.640
cg04406114	POU2F2	-137.53	316.57	0.664
cg19918116	DNAH10	-113.53	281.2	0.687
cg14222701	AMBRA1	134.09	359.9	0.710
cg00505318	UBA3	80.72	222.56	0.717
cg20540629	SKAP1	-181.98	511.39	0.722
cg15112362	ZFP36L1	-261.13	759.01	0.731
cg08311647	USP6NL	-87.15	260.71	0.738
cg08109625	PRKCZ	75.54	284.73	0.791
cg17679916		-39.94	243.62	0.870
cg09881793		-48.83	388.81	0.900
cg27412523	UTRN	28.21	313.57	0.928

Probes	Gene	B	SE	p
cg18159646	SNORD22; SNHG1	3.61	372.56	0.992

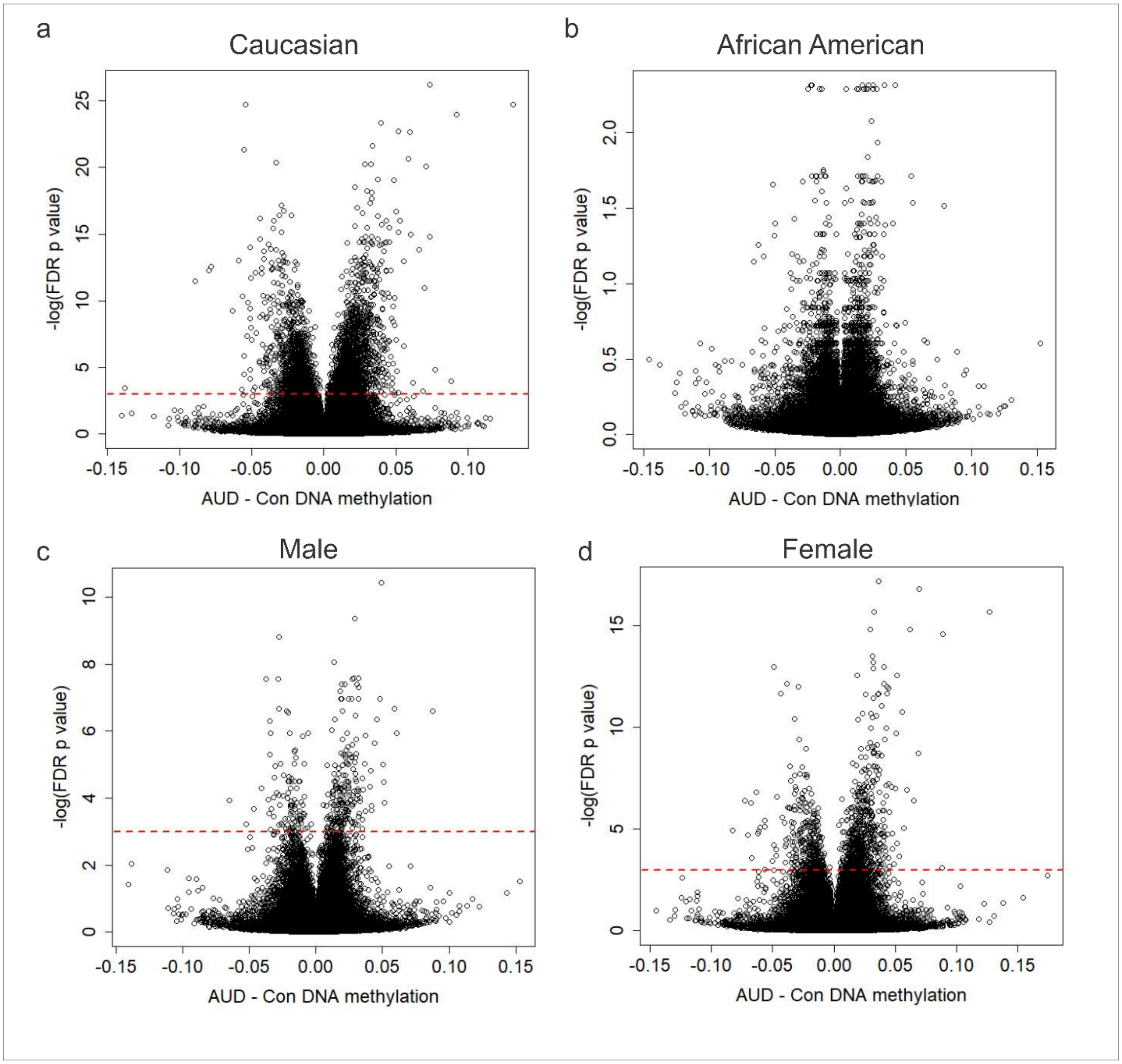

Supplementary Fig. 1 Volcano plots of DNA methylation association with AUD by ethnicity and gender. Volcano plots depicting the effect sizes of DNA methylation association with AUD (x axis) as a function of the negative natural log of the p value (y axis) for a) Caucasians, b) African American, c) Male, d) Female. Red dashed horizontal line depicts a nominal p value of 0.05.

Table S8. Associations between probe methylation and ethnicity and gender

Probes	Gene	B	SE	p
**More significant in Caucasians than African Americans**				
cg21095779	IL1RAP	0.047	0.0073	4.55E-06
cg00475490	PRSS23	0.025	0.0042	3.62E-05
cg16290996	GAS5;SNORD78	0.022	0.0038	7.38E-05
cg22818074	MAP2K1	-0.051	0.0089	7.99E-05
cg10558233		0.041	0.0071	8.79E-05
cg06644515	GAS5;SNORD81	0.022	0.0041	0.000215
cg08832418	SNORA51;SNORD110;NOP56	0.025	0.0047	0.000439
cg20005742		-0.033	0.0062	0.00054
cg26695233	LINC01126	0.02	0.0039	0.000709
cg06466940	ARRB1	0.015	0.0029	0.001202
cg22158648	CABLES1	0.02	0.0041	0.001415
cg19398525	ZNRF2	0.018	0.0037	0.001629
cg00666295	SNORD22;SNHG1	0.023	0.0049	0.002159
cg22998476		0.019	0.0039	0.002195
cg10204912	NFATC2	0.018	0.0039	0.003241
cg14377032	AUP1;HTRA2	0.024	0.0053	0.005113
cg08311647	USP6NL	0.018	0.0041	0.007922
cg25436965	TMEM72-AS1	0.019	0.0043	0.00913
cg05994753	LOC101927588	0.014	0.0035	0.021115
cg17679916		0.022	0.0055	0.021961
cg15356233		0.023	0.0056	0.024431
cg12726743	CAMK2G	0.014	0.0035	0.035391
cg27650500		0.012	0.0031	0.035401
cg14316231	MYST3	0.016	0.0041	0.041133
**More significant in African Americans than Caucasians**				
cg16562703	RNU6-71P;DST	0.013	0.0041	0.002299
cg27412523	UTRN	0.017	0.0055	0.002319
cg09881793		0.009	0.0033	0.004464
cg03883939	AMIGO3RNF123	-0.014	0.0048	0.004589
cg23010288		0.014	0.0051	0.005909
cg09095403	HPS4	-0.009	0.0036	0.010236
cg03401021	LINC01599	0.006	0.0024	0.012605
cg18696027	B2M	0.009	0.0035	0.015473
cg15554126	PEX14	-0.011	0.0046	0.017413
cg08109625	PRKCZ	-0.01	0.0043	0.02353
cg12169251	MYH9	0.009	0.0038	0.023918
cg05299572	C1orf56;BNIPL	0.008	0.0033	0.025307
cg00238470	TRAPPC3	0.008	0.0034	0.025613
cg16825803		0.012	0.0054	0.032694
**More significant in females than males**				
cg11095027	TOLLIP	-0.029	0.0043	6.28E-06
cg00501876	CSRNP1	0.026	0.0039	9.13E-06
cg02517348		0.034	0.0058	0.000307
cg18268027	ITPR1	0.025	0.0045	0.000836
cg20184330	ELMSAN1	0.027	0.005	0.001652

Probes	Gene	B	SE	p
cg18752527	HECW2	0.011	0.0022	0.002633
cg21095779	IL1RAP	0.039	0.0076	0.002703
cg25001882		0.02	0.0039	0.002755
cg21108805	WIF1	0.029	0.0057	0.002762
cg21053786		0.024	0.0048	0.003749
cg27537125		0.015	0.0031	0.003837
cg07267541	LURAP1L;LURAP1L-AS1	0.033	0.0068	0.006952
cg17825846		0.023	0.0047	0.009038
cg00475490	PRSS23	0.023	0.0048	0.009636
cg14391737	PRSS23	0.04	0.0085	0.0098
cg23999572	LINC-PINT	0.03	0.0063	0.010285
cg20732869	SLC1A2	0.022	0.0048	0.011821
cg07687574	PIK3R5	0.018	0.0041	0.016099
cg03603381	RASGRP1	0.02	0.0043	0.016318
cg14377032	AUP1;HTRA2	0.024	0.0054	0.022684
cg10558233		0.036	0.0082	0.027221
cg26952862		0.014	0.0033	0.028235
cg09975715	MYH10	-0.021	0.005	0.037358
cg19918116	DNAH10	0.017	0.0041	0.039951
cg27650500		0.013	0.0032	0.040509
cg16562703	RNU6-71P;DST	0.016	0.0038	0.046389
cg24797066		0.013	0.0031	0.047917

Table S9. AUD-associated co-regulated module loci derived from weighted genome coregulation network analysis (WGCNA)

ILMNID	Genes	AUD Effect WGCNA	AUD WGCNA P value	Module Membership	Module Membership P value
cg01940273		-0.41647	5.02E-24	0.692734	2.94E-78
cg05575921	*AHRR*	-0.4116	1.88E-23	0.672907	2.51E-72
cg21566642	*CABLES1*	-0.40601	8.30E-23	0.692335	3.92E-78
cg02583484	*HNRNPA1*	-0.38688	1.09E-20	0.735386	8.30E-93
cg17739917	*RARA*	-0.37111	4.82E-19	0.664008	8.19E-70
cg09511513	*ATP6V1B2*	-0.35109	4.43E-17	0.719607	4.14E-87
cg18387338		-0.30673	3.32E-13	0.563112	2.04E-46
cg08230167	*LURAP1L-AS1*	-0.29669	2.05E-12	0.571776	4.10E-48
cg07267541	*LURAP1L*	-0.29498	2.78E-12	0.655868	1.37E-67
cg10558233		-0.29158	5.04E-12	0.55751	2.41E-45
cg26952862	*UTRN*	-0.2723	1.28E-10	0.60646	1.92E-55
cg18917643		-0.27186	1.38E-10	0.533844	4.90E-41
cg21095779	*WIF1*	-0.26648	3.25E-10	0.577588	2.79E-49
cg21108805		-0.26175	6.80E-10	0.587961	2.01E-51
cg21053786		-0.25683	1.44E-09	0.644499	1.36E-64
cg20813374	*FKBP5*	-0.25611	1.61E-09	0.567624	2.71E-47
cg20625334	*STAM2*	-0.25419	2.15E-09	0.519528	1.37E-38
cg17825846		-0.24897	4.65E-09	0.677757	9.81E-74
cg03603381	*RASGRP1*	-0.23988	1.71E-08	0.43827	1.05E-26
cg25001882		-0.23583	3.01E-08	0.566348	4.81E-47
cg16290996	*GAS5*	-0.23462	3.56E-08	0.604733	4.67E-55
cg08832418	*SNORA51*	-0.23231	4.88E-08	0.525635	1.28E-39
cg15356233		-0.22507	1.28E-07	0.408549	4.24E-23
cg15408734		-0.2238	1.52E-07	0.427583	2.28E-25
cg02517348		-0.22133	2.09E-07	0.446662	8.61E-28
cg16562703	*RNU6-71P*	-0.21459	4.93E-07	0.536716	1.53E-41
cg27372654		-0.20548	1.50E-06	0.330154	3.58E-15
cg25615944		-0.20375	1.85E-06	0.395986	1.11E-21
cg15251675		-0.1902	8.74E-06	0.400459	3.53E-22
cg21021065		-0.18129	2.29E-05	0.535494	2.52E-41
cg20725067	*UBE2E1*	-0.17066	6.83E-05	0.314987	7.03E-14
cg25537245	*LINC01126*	-0.17007	7.24E-05	0.418685	2.74E-24
cg05994753	*LOC101927588*	-0.16956	7.62E-05	0.509134	6.91E-37
cg15130459	*SNORD72*	-0.16876	8.25E-05	0.357826	1.00E-17
cg13683827	*FAM113B*	-0.16498	0.000119	0.501762	1.03E-35
cg14316231	*MYST3*	-0.15388	0.000336	0.402785	1.93E-22

ILMNID	Genes	AUD Effect WGCNA	AUD WGCNA P value	Module Membership	Module Membership P value
cg12792732	*ATP6V1H*	-0.12519	0.003602	0.162705	0.000148
cg26695233	*MEF2C*	-0.12296	0.004252	0.261479	7.09E-10
cg18696027	*B2M*	-0.12293	0.004261	0.238502	2.08E-08
cg22158648		-0.10181	0.018062	0.279632	3.86E-11
cg22140810	*MAP2K1*	0.199529	3.03E-06	-0.32676	7.08E-15
cg15554126	*PEX14*	0.201187	2.50E-06	-0.30719	3.05E-13
cg03883939	*AMIGO3*	0.210538	8.14E-07	-0.42168	1.20E-24
cg11589723	*GLTSCR1*	0.223145	1.65E-07	-0.28453	1.70E-11
cg20005742		0.235294	3.24E-08	-0.29645	2.14E-12
cg09975715	*MYH10*	0.295468	2.55E-12	-0.47432	1.37E-31
cg18581607	*PLD2*	0.339264	5.51E-16	-0.60493	4.23E-55
cg15627993	*LDLRAD4*	0.356526	1.34E-17	-0.60527	3.55E-55
cg02606535	*TSC2*	0.357679	1.04E-17	-0.3598	6.44E-18
cg11095027	*TOLLIP*	0.36475	2.10E-18	-0.47872	3.17E-32

Table S10. Probes showing significant associations with task-based fMRI BOLD signals and their overlap with probes associated with AUD status

Target probes associated with AUD, methylation and BOLD signal		Discovery cohort (n = 539)			First blood replication cohort (n = 86)			Correlations between methylation and BOLD signal (data from 1st blood replication cohort, n = 86)		
**CpG site**	**Gene**	**B**	**SE**	**p**	**B**	**SE**	**p**	**Brain region**	**Kendall's tau**	**p**
1.cg25436965	*TMEM72-AS1*	-0.014	0.003	0.007	-0.044	0.008	0.013	Left Amygdala	-0.150	0.040591
2.cg05575921	*AHRR*	-0.104	0.011	<0.001	-0.144	0.024	0.004	Right Amygdala	0.177	0.015651
3.cg21053786	–	-0.020	0.003	<0.001	-0.044	0.009	0.031	Right Amygdala	0.146	0.045585
4.cg10558233	–	-0.026	0.005	0.001	-0.066	0.013	0.031	Right Amygdala	0.144	0.04891
5.cg08230167	*LURAP1L*	-0.034	0.004	<0.001	-0.055	0.011	0.031	Right Amygdala	0.217	0.002988
6.cg12169251	*MYH9*	-0.010	0.003	0.038	-0.028	0.006	0.037	Right Amygdala	0.147	0.044785
7.cg17739917	*RARA*	-0.047	0.005	<0.001	-0.069	0.011	0.002	Right Amygdala	0.170	0.020342
8.cg09975715	*MYH10*	0.019	0.003	<0.001	0.042	0.008	0.028	Left Insula	0.154	0.035737
9.cg03603381	*RASGRP1*	-0.015	0.003	0.001	-0.038	0.008	0.038	Left Insula	-0.158	0.030232
								Right Insula	-0.175	0.01681
10.cg15705813	–	-0.021	0.003	<0.001	-0.038	0.006	0.004	Left Prefrontal Cortex	-0.195	0.007828
								Right Prefrontal Cortex	-0.183	0.012194
								Bilateral Prefrontal Cortex	-0.153	0.036066

Target probes associated with AUD, methylation and BOLD signal		Discovery cohort (n = 539)			First blood replication cohort (n = 86)			Correlations between methylation and BOLD signal (data from 1st blood replication cohort, n = 86)		
CpG site	Gene	B	SE	p	B	SE	p	Brain region	Kendall's tau	p
11.cg08109625	*PRKCZ*	0.011	0.003	0.044	0.036	0.007	0.023	Left Prefrontal Cortex	0.192	0.00855
								Right Prefrontal Cortex	0.259	0.000395
								Bilateral Prefrontal Cortex	0.181	0.013542
12.cg14222701	*AMBRA1*	0.011	0.002	0.002	0.033	0.006	0.006	Left Prefrontal Cortex	0.205	0.005093
								Right Prefrontal Cortex	0.224	0.00217
								Right Hippocampus	0.231	0.001623
13.cg00475490	*PRSS23*	-0.017	0.003	0.001	-0.042	0.008	0.031	Left Prefrontal Cortex	-0.154	0.035737
								Bilateral Prefrontal Cortex	-0.161	0.027763
14.cg20813374	*FKBP5*	-0.020	0.004	<0.001	-0.045	0.009	0.030	Left Prefrontal Cortex	-0.155	0.034445
								Right Prefrontal Cortex	-0.228	0.001866
								Bilateral Prefrontal Cortex	-0.193	0.008272
15.cg19918116	*DNAH10*	-0.015	0.003	<0.001	-0.031	0.006	0.030	Left Prefrontal Cortex	-0.184	0.011814
								Right Prefrontal Cortex	-0.172	0.018783
								Bilateral Prefrontal Cortex	-0.219	0.002777
16.cg25537245	–	-0.006	0.001	0.010	-0.016	0.003	0.049	Right Prefrontal Cortex	-0.187	0.010507

Target probes associated with AUD, methylation and BOLD signal		Discovery cohort (n = 539)			First blood replication cohort (n = 86)			Correlations between methylation and BOLD signal (data from 1st blood replication cohort, n = 86)		
**CpG site**	**Gene**	**B**	**SE**	**p**	**B**	**SE**	**p**	**Brain region**	**Kendall's tau**	**p**
								Bilateral Prefrontal Cortex	-0.174	0.017329
17.cg08832418	*NOP56*	-0.018	0.004	0.001	-0.048	0.009	0.013	Right Prefrontal Cortex	-0.145	0.047223
								Bilateral Prefrontal Cortex	-0.161	0.027763
18.cg04406114	*POU2F2*	0.010	0.002	0.003	0.024	0.005	0.034	Right Prefrontal Cortex	0.174	0.017683
19.cg15627993	*LDLRAD4*	0.034	0.005	<0.001	0.059	0.012	0.028	Right Prefrontal Cortex	0.144	0.04891
								Bilateral Prefrontal Cortex	0.189	0.009849
20.cg17825846	–	-0.017	0.003	<0.001	-0.038	0.007	0.023	Right Prefrontal Cortex	-0.163	0.025717
21.cg18268027	*ITPR1*	-0.021	0.003	<0.001	-0.045	0.009	0.020	Right Prefrontal Cortex	-0.152	0.038097
								Right Hippocampus	-0.166	0.023571
22.cg10204912	*NFATC2*	-0.013	0.003	0.007	-0.035	0.007	0.022	Right Prefrontal Cortex	-0.166	0.023344
								Bilateral Prefrontal Cortex	-0.151	0.039505
23.cg20184330	*ELMSAN1*	-0.022	0.003	<0.001	-0.038	0.008	0.037	Bilateral Prefrontal Cortex	-0.145	0.048061
24.cg27537125	–	-0.013	0.002	<0.001	-0.024	0.004	0.012	Bilateral Prefrontal Cortex	-0.150	0.040225
25.cg26952862	–	-0.014	0.002	<0.001	-0.029	0.006	0.030	Bilateral Prefrontal Cortex	-0.159	0.029947
26.cg07687574	*PIK3R5*	-0.015	0.003	<0.001	-0.035	0.007	0.031	Bilateral Prefrontal Cortex	-0.193	0.008456

Target probes associated with AUD, methylation and BOLD signal		Discovery cohort (n = 539)			First blood replication cohort (n = 86)			Correlations between methylation and BOLD signal (data from 1st blood replication cohort, n = 86)		
**CpG site**	**Gene**	**B**	**SE**	**p**	**B**	**SE**	**p**	**Brain region**	**Kendall's tau**	**p**
27.cg18581607	*PLD2*	0.036	0.006	<0.001	0.071	0.014	0.028	Right Hippocampus	0.160	0.028564
28.cg27650500	–	-0.008	0.002	0.043	-0.032	0.006	0.010	Right Hippocampus	-0.149	0.042073
29.cg18752527	*HECW2*	-0.007	0.001	0.001	-0.017	0.003	0.020	Right Anterior Cingulate Cortex	0.164	0.024506

Table S11. Associations of methylation at the target 96 probes with alcohol use status in postmortem samples

		First Brain replication cohort (n = 58)		
**Probe**	**Gene**	**Neuronal tissue**		
		**B**	**SE**	**p**
cg20005742		-0.065	0.020	0.002
cg06644515	*GAS5*	0.023	0.009	0.014
cg11589723	*GLTSCR1*	-0.030	0.014	0.041
cg18696027	*B2M*	-0.009	0.004	0.051
cg22158648	*CABLES1*	-0.036	0.019	0.073
cg22998476		0.007	0.004	0.093
cg04406114	*POU2F2*	-0.017	0.011	0.118
cg20625334	*STAM2*	0.008	0.006	0.138
cg25615944	*RPL23*	0.023	0.016	0.164
cg15130459	*RPL37*	0.021	0.017	0.241
cg05575921	*AHRR*	0.007	0.006	0.245
cg18752527	*HECW2*	-0.010	0.010	0.294
cg08109625	*PRKCZ*	-0.010	0.010	0.327
cg20813374	*FKBP5*	0.004	0.005	0.458
cg16290996	*GAS5*	0.006	0.008	0.483
cg14334350	*NOTCH1*	0.004	0.007	0.507
cg14316231	*MYST3*	0.005	0.008	0.543
cg02583484	*HNRNPA1*	0.007	0.012	0.555
cg00505318	*UBA3*	0.003	0.005	0.557
cg09975715	*MYH10*	-0.007	0.018	0.689
cg21566642		-0.004	0.011	0.704
cg01940273		0.003	0.008	0.742
cg11095027	*TOLLIP*	-0.004	0.013	0.752
cg15408734		-0.002	0.009	0.817
cg00501876	*CSRNP1*	-0.003	0.015	0.842
cg18159646	*SNORD30, SNHG1*	-0.001	0.015	0.952
cg15554126	*PEX14*	0.000	0.006	0.999
**Probe**	**Gene**	**Glial tissue**		
		**B**	**SE**	**p**
cg08109625	*PRKCZ*	-0.013	0.006	0.040
cg05575921	*AHRR*	0.023	0.013	0.088
cg16290996	*GAS5*	0.021	0.012	0.090
cg18696027	*B2M*	0.013	0.009	0.142
cg14316231	*MYST3*	0.019	0.013	0.150
cg15130459	*RPL37*	0.021	0.016	0.192
cg00505318	*UBA3*	0.020	0.015	0.200
cg04406114	*POU2F2*	0.007	0.006	0.208
cg22158648	*CABLES1*	-0.023	0.020	0.243
cg14334350	*NOTCH1*	-0.009	0.008	0.248
cg15554126	*PEX14*	-0.008	0.007	0.283
cg21566642		-0.010	0.010	0.326
cg22998476		-0.004	0.004	0.331
cg00501876	*CSRNP1*	-0.014	0.021	0.503

cg25615944	*RPL23*	0.014	0.021	0.506
cg18159646	*SNORD30, SNHG1*	-0.007	0.012	0.542
cg20005742		0.016	0.027	0.544
cg01940273		0.006	0.010	0.553
cg02583484	*HNRNPA1*	0.006	0.011	0.588
cg18752527	*HECW2*	0.007	0.013	0.627
cg20625334	*STAM2*	-0.003	0.008	0.682
cg06644515	*GAS5*	0.004	0.016	0.791
cg20813374	*FKBP5*	0.003	0.010	0.796
cg11095027	*TOLLIP*	0.002	0.008	0.808
cg09975715	*MYH10*	-0.002	0.009	0.822
cg15408734		0.001	0.007	0.917
cg11589723	*GLTSCR1*	0.000	0.006	0.996

Second brain replication cohort (GSE49393)
(n = 48)

Probe	Gene	B	SE	p
cg00505318	*UBA3*	0.033	0.010	0.002
cg15705813		-0.028	0.009	0.003
cg22158648	*CABLES1*	0.025	0.009	0.008
cg14316231	*MYST3*	-0.031	0.012	0.011
cg06644515	*GAS5*	0.022	0.009	0.015
cg10558233		-0.033	0.013	0.016
cg09975715	*MYH10*	0.017	0.007	0.018
cg11095027	*TOLLIP*	0.021	0.008	0.018
cg02583484	*HNRNPA1*	0.014	0.006	0.024
cg08109625	*PRKCZ*	-0.022	0.010	0.037
cg11589723	*GLTSCR1*	0.022	0.011	0.043
cg04406114	*POU2F2*	-0.010	0.005	0.058
cg05575921	*AHRR*	-0.013	0.007	0.064
cg20005742		0.031	0.017	0.077
cg25537245		-0.019	0.011	0.080
cg18752527	*HECW2*	-0.017	0.010	0.093
cg15408734		0.004	0.002	0.093
cg15554126	*PEX14*	-0.006	0.004	0.123
cg14334350	*NOTCH1*	0.007	0.005	0.138
cg21566642		-0.009	0.006	0.161
cg18696027	*B2M*	-0.009	0.006	0.170
cg20813374	*FKBP5*	-0.014	0.010	0.176
cg16290996	*GAS5*	0.008	0.007	0.261
cg00501876	*CSRNP1*	-0.009	0.008	0.265
cg22998476		0.002	0.002	0.282
cg13683827	*FAM113B*	-0.010	0.011	0.395
cg18159646	*SNORD30,SNHG1*	0.005	0.007	0.452
cg18917643		-0.008	0.010	0.453
cg27537125		0.004	0.005	0.461
cg20625334	*STAM2*	-0.003	0.006	0.541
cg15130459	*RPL37*	0.004	0.009	0.631
cg03603381	*RASGRP1*	-0.003	0.006	0.658
cg25615944	*RPL23*	-0.004	0.008	0.662
cg01940273		0.000	0.005	0.972

Table S12. Results of gene ontology analysis of the 96 FDR corrected CpG sites identified from the discovery and first replication cohorts

GO Category	N identified	N in group	FDR adjusted p-value	Function
GO:0071705	23	2369	0.0003	nitrogen compound transport
GO:0015031	20	2007	0.002	protein transport
GO:0006810	33	5072	0.002	transport
GO:0015833	20	2043	0.003	peptide transport
GO:0042886	20	2073	0.003	amide transport
GO:0051234	33	5187	0.004	establishment of localization
GO:0045184	20	2113	0.005	establishment of protein localization
GO:0051649	20	2142	0.006	establishment of localization in cell
GO:0032633	4	34	0.006	interleukin-4 production
GO:0071702	23	2790	0.006	organic substance transport
GO:0051641	23	2804	0.007	cellular localization
GO:0009611	11	665	0.009	response to wounding
GO:0033036	24	3088	0.010	macromolecule localization
GO:0032754	3	13	0.014	positive regulation of interleukin-5 production
GO:0032736	3	14	0.018	positive regulation of interleukin-13 production
GO:0008104	22	2737	0.018	protein localization
GO:0043009	10	587	0.020	chordate embryonic development
GO:0048534	12	872	0.021	hematopoietic or lymphoid organ development
GO:0009792	10	607	0.027	embryo development ending in birth or egg hatching
GO:0060324	4	49	0.028	face development
GO:0051049	17	1783	0.031	regulation of transport
GO:0051128	20	2397	0.032	regulation of cellular component organization
GO:0055012	3	17	0.033	ventricular cardiac muscle cell differentiation
GO:0051179	36	6563	0.034	localization
GO:0002520	12	918	0.035	immune system development
GO:0002521	9	505	0.041	leukocyte differentiation

References Supplementary:

1. Meyer, M.A. Highly Expressed Genes within Hippocampal Sector CA1: Implications for the Physiology of Memory. *Neurol Int* **6**, 5388-5388 (2014).
2. Shahani, N.*, et al.* RasGRP1 promotes amphetamine-induced motor behavior through a Rhes interaction network ("Rhesactome") in the striatum. *Science Signaling* **9**, ra111 (2016).
3. Haberman, R.P., Lee, H.J., Colantuoni, C., Koh, M.T. & Gallagher, M. Rapid encoding of new information alters the profile of plasticity-related mRNA transcripts in the hippocampal CA3 region. *Proceedings of the National Academy of Sciences* **105**, 10601 (2008).
4. Hamdan, F.F.*, et al.* De Novo Mutations in Moderate or Severe Intellectual Disability. *PLOS Genetics* **10**, e1004772 (2014).
5. Ozkan, E.D.*, et al.* Input-specific regulation of hippocampal circuit maturation by non-muscle myosin IIB. *Journal of Neurochemistry* **134**, 429-444 (2015).
6. Brückmann, C.*, et al.* DNA methylation signatures of chronic alcohol dependence in purified CD3+ T-cells of patients undergoing alcohol treatment. *Scientific Reports* **7**, 6605 (2017).
7. Parker, S.S.*, et al.* Competing molecular interactions of aPKC isoforms regulate neuronal polarity. *Proceedings of the National Academy of Sciences* **110**, 14450 (2013).
8. Wang, S., Sheng, T., Ren, S., Tian, T. & Lu, W. Distinct Roles of PKCι/λ and PKMζ in the Initiation and Maintenance of Hippocampal Long-Term Potentiation and Memory. *Cell Reports* **16**, 1954-1961 (2016).
9. Flanagan, J.M.*, et al.* Temporal stability and determinants of white blood cell DNA methylation in the breakthrough generations study. *Cancer epidemiology, biomarkers & prevention : a publication of the American Association for Cancer Research, cosponsored by the American Society of Preventive Oncology* **24**, 221-229 (2015).
10. Palmisano, M. & Pandey, S.C. Epigenetic mechanisms of alcoholism and stress-related disorders. *Alcohol* **60**, 7-18 (2017).
11. Alicia K Smith, A.R., Adam X Maihofer, Robert K Naviaux, Allison E Aiello, Ananda B Amstadter, Allison E Ashley-Koch, Dewleen G Baker, Jean C Beckham, Marco P Boks, Evelyn Bromet, Michelle Dennis, Sandro Galea, Melanie E Garrett, Elbert Geuze, Guia Guffanti, Michael A Hauser, Seyma Katrinli, Varun Kilaru, Ronald C. Kessler, Nathan A Kimbrel, Karestan C Koenen, Pei-Fen Kuan, Kefeng Li, Mark W Logue, Adriana Lori, Benjamin J Luft, Mark W Miller, Jane C Naviaux, Nicole R Nugent, Xuejun Qin, Kerry J Ressler, Victoria B Risbrough, Bart P F Rutten, Murray B Stein, Robert J. Ursano, Eric Vermetten, Christiaan H Vinkers, Lin Wang, Nagy A Youssef, INTRuST Clinical Consortium, VA Mid-Atlantic MIRECC Workgroup, PGC PTSD Epigenetics Workgroup, Monica Uddin, Caroline M Nievergelt. Epigenome-wide meta-analysis of PTSD across 10 military and civilian cohorts identifies novel methylation loci. ((https://www.biorxiv.org/content/10.1101/585109v1), 2019).
12. La Barbera, L., Vedele, F., Nobili, A., D'Amelio, M. & Krashia, P. Neurodevelopmental Disorders: Functional Role of Ambra1 in Autism and Schizophrenia. *Molecular Neurobiology* (2019).
13. Nobili, A.*, et al.* Ambra1 Shapes Hippocampal Inhibition/Excitation Balance: Role in Neurodevelopmental Disorders. *Molecular Neurobiology* **55**, 7921-7940 (2018).
14. Nguyen, T. & Di Giovanni, S. NFAT signaling in neural development and axon growth. *International Journal of Developmental Neuroscience* **26**, 141-145 (2008).

15. Jarvis, C.I., *et al.* Retinoic acid receptor-α signalling antagonizes both intracellular and extracellular amyloid-β production and prevents neuronal cell death caused by amyloid-β. *European Journal of Neuroscience* **32**, 1246-1255 (2010).
16. Boku, S., *et al.* Neonatal Maternal Separation Alters the Capacity of Adult Neural Precursor Cells to Differentiate into Neurons Via Methylation of Retinoic Acid Receptor Gene Promoter. *Biological Psychiatry* **77**, 335-344 (2015).
17. Chen, X.-N., *et al.* The Involvement of Retinoic Acid Receptor-α in Corticotropin-Releasing Hormone Gene Expression and Affective Disorders. *Biological Psychiatry* **66**, 832-839 (2009).
18. Kumar, A., Singh, C.K., DiPette, D.D. & Singh, U.S. Ethanol Impairs Activation of Retinoic Acid Receptors in Cerebellar Granule Cells in a Rodent Model of Fetal Alcohol Spectrum Disorders. *Alcoholism: Clinical and Experimental Research* **34**, 928-937 (2010).
19. Khachatoorian, R., *et al.* SAMe treatment prevents the ethanol-induced epigenetic alterations of genes in the Toll-like receptor pathway. *Experimental and Molecular Pathology* **94**, 243-246 (2013).
20. Humbert-Claude, M., *et al.* Tollip, an early regulator of the acute inflammatory response in the substantia nigra. *Journal of neuroinflammation* **13**, 303 (2016).
21. Kowalski, E., Geng, S., Rathes, A., Lu, R. & Li, L. Toll-interacting protein differentially modulates HIF1α and STAT5-mediated genes in fibroblasts. *Journal of Biological Chemistry* **293**, 12239-12247 (2018).
